# Histological and prognostic data on surgically resected early-stage lung adenocarcinoma

**DOI:** 10.1016/j.dib.2020.105785

**Published:** 2020-05-29

**Authors:** Masaya Yotsukura, Hisao Asamura, Shigeki Suzuki, Keisuke Asakura, Yukihiro Yoshida, Kazuo Nakagawa, Hiroyuki Sakurai, Shun-ichi Watanabe, Noriko Motoi

**Affiliations:** 1 Department of Pathology and Clinical Laboratories, National Cancer Center Hospital, 5-1-1 Tsukiji, Chuo-ku, Tokyo 104-0045, Japan; 2 Department of Thoracic Surgery, National Cancer Center Hospital, 5-1-1 Tsukiji, Chuo-ku, Tokyo 104-0045, Japan; 3 Division of Thoracic Surgery, Keio University School of Medicine, 35, Shinanomachi, Shinjuku-ku, Tokyo 160-8582, Japan; 4 Department of Thoracic Surgery, Sagamihara Kyodo Hospital, 2-8-18, Hashimoto, Midori-ku, Sagamihara, Kanagawa Prefecture 252-5188, Japan; 5 Division of Respiratory Surgery, Nihon University School of Medicine, 30-1 Oyaguchikamimachi, Itabashi-ku, Tokyo 173-8610, Japan; 6 Department of Diagnostic Pathology, National Cancer Center Hospital, 5-1-1 Tsukiji, Chuo-ku, Tokyo 104-0045, Japan

**Keywords:** Lung adenocarcinoma, histological subtype, cancer-associated active fibroblast, recurrence

## Abstract

This article presents supplementary data for the research article by Yotsukura et al. entitled “Prognostic impact of cancer-associated active fibroblasts and invasive architectural patterns on early-stage lung adenocarcinoma” [1], which presented the postoperative prognosis for early-stage lung adenocarcinoma categorized according to histological findings. We included data of 1,032 resected cases of lung adenocarcinoma, which consisted of pathological stage IA invasive cancer and adenocarcinoma in situ resected at National Cancer Center Hospital, Tokyo, Japan, between 2007 and 2012. A pathological review was performed to assess total tumor size, size of invasion, histological subtype, lymphovascular invasion, and presence of cancer-associated active fibroblast (CAF). Tumor recurrence and overall survival were retrospectively recorded. Of the included cases, 166 (16.1%), and 866 (83.9%) were adenocarcinoma in situ and pathological stage IA, respectively. Pathological stage IA adenocarcinoma was further classified based on the histologial subtype and the presence of CAF. This data set may be useful for analyzing the postoperative prognosis of early-stage lung adenocarcinoma, in combination with detailed pathological findings including size of invasion, histological subtype, and presence of CAF.

Specifications table

**Table:** 

Subject	Oncology

Specific subject area	Postoperative prognosis of early-stage lung adenocarcinoma with histological information
Type of data	Table, Fig.s
How data were acquired	The data were acquired from a survey of medical records and microscopic findings of surgically resected specimens at National Cancer Center Hospital, Tokyo, Japan.
Data format	Raw and partially filtered
Parameters for data collection	The data consist of pathological findings of stage IA lung adenocarcinoma and adenocarcinoma in situ, including total tumor diameter, size of invasion, lymphovascular invasion, and histological subtype. The data also show clinical information including age, gender, operative procedure, tumor location, and prognosis.
Description of data collection	The data were acquired from 1,032 cases of pathological stage IA lung adenocarcinoma and adenocarcinoma in situ who underwent complete resection between 2007 and 2012 at National Cancer Center Hospital, Tokyo, Japan. A pathological review was performed to assess total tumor size, size of invasion, histological subtype, lymphovascular invasion, and presence of cancer-associated active fibroblast (CAF). Age, gender, tumor recurrence and overall survival status were collected from medical records.
Data source location	5-1-1, Tsukiji, Chuo-ku, Tokyo, Japan
Data accessibility	The data are presented with the article.
Related research article	Masaya Yotsukura, Hisao Asamura, Shigeki Suzuki, Keisuke Asakura, Yukihiro Yoshida, Kazuo Nakagawa, Hiroyuki Sakurai, Shun-ichi Watanabe, Noriko Motoi. Prognostic impact of cancer-associated active fibroblasts and invasive architectural patterns on early-stage lung adenocarcinoma. Lung Cancer. In Press

## Value of the data

• The data provide detailed pathological findings of early-stage lung adenocarcinoma based on the 2015 World Health Organization histological classification in combination with the long-term postoperative prognosis.
• The data should be useful for analyzing the prognostic value of invasive size and histological subtype of lung adenocarcinoma.
• Studies on the surgical oncology of lung adenocarcinoma could use the data to analyze the relationships among clinical and pathological findings and the long-term postoperative prognosis.
• The detailed classification of histological subtype and presence of cancer-associated active fibroblasts will also allow researchers to conduct detailed studies on the prognosis of adenocarcinoma with very early-phase of invasion.

## 1 Data Description

**Fig. 1:**
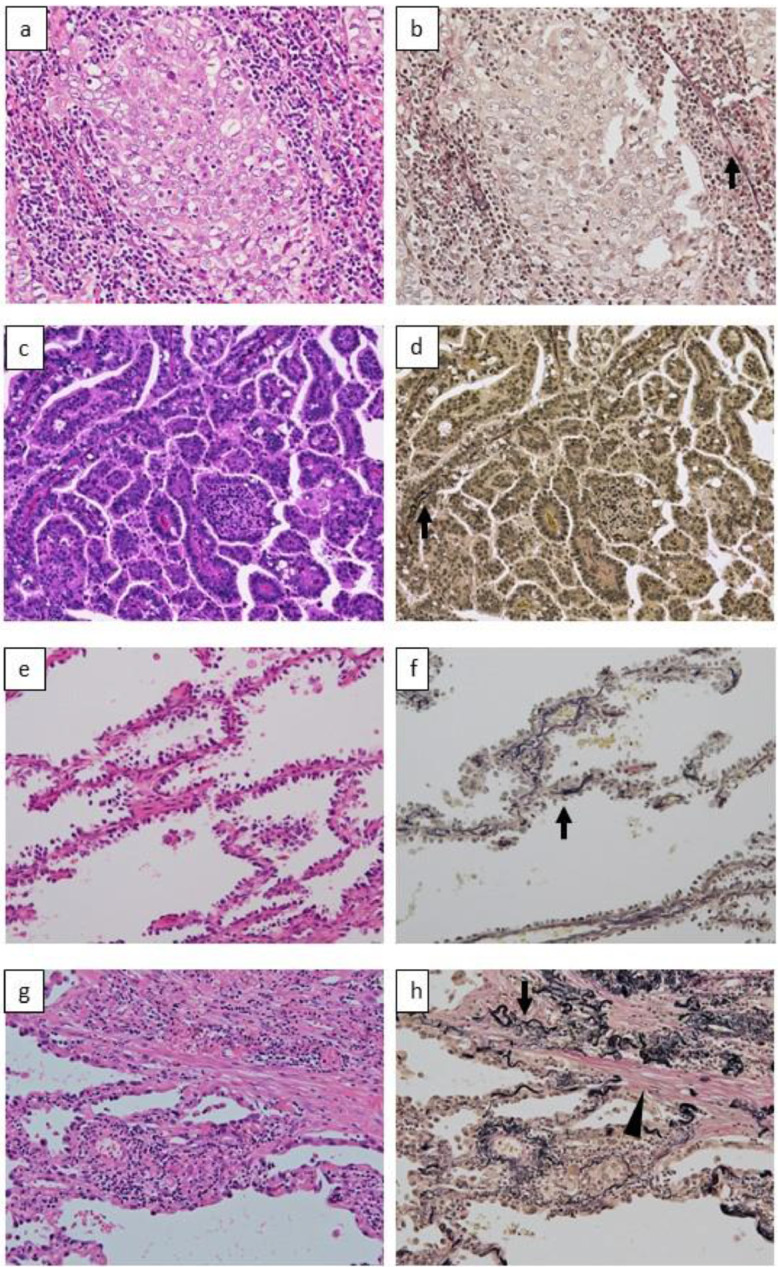
Representative microscopic images of solid-pattern adenocarcinoma **(a, b)**, papillary-pattern adenocarcinoma **(c, d)**, adenocarcinoma in situ (AIS) **(e, f)**, and INV-2 tumor **(g, h)**. Solid-pattern adenocarcinoma shows sheet-like growth of tumor cells, with destroyed elastic fibers of the remnant alveolus around the tumor nest (arrow) **(a, b)**. Papillary-pattern adenocarcinoma shows secondary-to-tertiary papillae in which glandular cells grow along with the fibrovascular core independent of the surrounding alveolar elastic fibers (arrow) **(c, d)**. AIS shows the proliferation of tumor cells in a monolayer fashion along the surface of the alveolar walls **(e)**. Elastica van Gieson staining reveals the preserved alveolar elastic fibers (arrow) **(f)**. In INV-2 adenocarcinoma, tumor cells grow in a monolayer fashion along the surface of alveolar walls, and active fibroblasts (arrowhead) are present in the thickened alveolar interstitial tissue **(g)**. Alveolar elastic fibers are observed as a single thin line or as a dense aggregation (arrow) along the tumor cells **(h)**. Original magnification: x100 in all pictures. Hematoxylin-eosin (a, c, e, g) and Elastica van Gieson (b, d, f, h) staining.

**Fig. 2:**
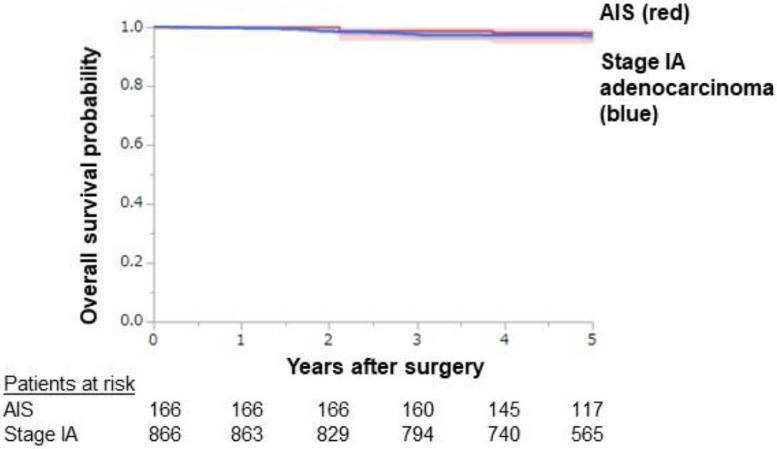
Kaplan–Meier curves for overall survival with 95% confidence intervals for pathological stage IA adenocarcinoma and adenocarcinoma in situ cases. The 5-year overall survival rates of stage IA adenocarcinoma and AIS were 97.0% (95% CI: 95.8-98.1) and 98.1% (95% CI: 96.0-100), respectively.

Fig. 1 shows representative pathological findings of solid adenocarcinoma, papillary adenocarcinoma, adenocarcinoma in situ (AIS), and lepidic adenocarcinoma with cancer-associated fibroblasts, stained by hematoxylin and eosin (HE) and Elastica–van Gieson (EVG). Fig. 2 shows overall survival curves of stage IA adenocarcinoma and AIS depicted by the Kaplan-Meier method. The estimated 3- /5-year overall survival rates were 97.4% (95% confidence interval [CI]: 96.3-98.5)/97.0% (95% CI: 95.8-98.1) and 98.8% (95% CI: 97.1-100)/98.1% (95% CI: 96.0-100) for stage IA cancer and AIS, respectively. The numbers of patients at risk were 866/166 at the time of the operation, 794/160 at postoperative year 3, and 565/117 at postoperative year 5 for stage IA adenocarcinoma/AIS, respectively.

**Supplementary Table** shows raw data of the enrolled 1,032 cases. The data include age, gender, smoking history, operative procedure, tumor location, follow-up duration, survival and recurrence status, pathological total tumor diameter, size of invasion, histological subtype, and lymphovascular invasion. Histological subtype was determined according to the 4^th^ edition of the World Health Organization (WHO) classification of lung tumor [2].

## 2 Experimental Design, Materials, and Methods

Of the 2,393 patients who underwent resection for lung cancer at National Cancer Center Hospital, Tokyo, Japan, between January 2007 and December 2012, we collected the records of 1,032 patients with AIS or pathological stage IA adenocarcinoma who had undergone curative surgical resection, without a history of previous lung resection for a different lung cancer or a follow-up period of less than one year without recurrence. The pathological stage was assessed according to the 8^th^ edition of the TNM classification of the Union for International Cancer Control Classification [3].

In principle, we checked the recurrence of tumor by routine postoperative follow-up, using physical examination, blood analysis, and chest radiography or computed tomography, twice a year. Patients with suspicious symptoms or signs of recurrence were further evaluated using additional computed tomography, magnetic resonance imaging, positron emission tomography, and/or bone scintigraphy. Tumor recurrence was diagnosed according to the results of the above-mentioned evaluations. Follow-up evaluations were performed until the end of March 2019.

Surgically resected specimens were fixed using 10% neutral buffered formalin. Formalin was injected from the bronchial stump for cases of pneumonectomy, bilobectomy, lobectomy and segmentectomy. For cases of wedge resection, formalin was injected from the pleural side. Lung was inflated by injected formalin. Fixed specimens were cut serially into 5-mm-thick sections, then macroscopically sampled for paraffin-embedded blocks. Next, 4-µm-thick sections were cut and stained by HE and EVG.

Pathological re-evaluation was performed by two authors (MY, NM). Histological classification was determined based on the 4^th^ edition of the WHO classification [2]. Total tumor diameter, size of invasive component, lymphatic permeation, vascular invasion, pleural invasion, and histological subtype were evaluated. For ambiguous cases regarding lymphovascular invasion, we performed immunohistochemical staining for a lymphatic endothelial marker (Clone D2-40, Biolegend, San Diego, CA, USA) and a vascular endothelial marker (CD31, Clone JC70A, DAKO, Glostrup, Denmark).

Using the WHO criteria for the invasion of lung adenocarcinoma, we divided invasive tumors into two subgroups, INV-1 and INV-2. INV-1 was defined as an adenocarcinoma with a histological subtype other than a lepidic pattern (i.e., papillary, acinar, micropapillary, and/or solid), irrespective of the presence or absence of CAF. Meanwhile, INV-2 was defined as lepidic-pattern adenocarcinoma with the presence of CAF. The presumed invasive component in INV-2 was only CAF. Cases that showed a combination of lepidic and non-lepidic histological subtypes were classified as INV-1.

Based on the WHO criteria, a lepidic growth pattern was described as a cellular proliferation of pneumocytes along the surface of the alveolar walls [2]. When tumor cells grew regardless of the pre-existing alveolar structure, these cells were considered to have non-lepidic invasive patterns. Acinar pattern consisted of round to oval-shaped malignant glands invading a fibrous stroma. Papillary pattern showed malignant cuboidal to columnar tumor cells growing on the surface of fibrovascular cores. Solid pattern had sheets of proliferated tumor cells with abundant eosinophilic cytoplasm. Micropapillary pattern consisted of tumor cells growing in papillary tufts forming florets that lack fibrovascular cores. As an aid to histological evaluation, EVG staining was used to identify pre-existing alveolar elastic fiber. When it was difficult to determine the histological subtype, we considered it to be non-lepidic histological pattern if the tumor cells grew regardless of the alveolar elastic fiber, or if they destroyed architectural elastic fibers. Representative histological findings in AIS, papillary adenocarcinoma, solid adenocarcinoma, and lepidic adenocarcinoma with CAF (INV-2 tumor) are shown in Fig. 1.

The Kaplan-Meier method was used to evaluate the survival curves. The overall survival probability was assessed using the time from surgery until death due to any cause, or latest follow-up date without death. Statistical analyses were performed using JMP® 15 (SAS Institute Inc., Cary, NC, USA).

## Ethics Statement

The study protocol was approved by the Medical Research Ethics Committee of the National Cancer Center (IRB approval No. 2015-289), and all experiments were conducted in accordance with the Declaration of Helsinki. The requirement for informed consent was waived by the committee since our study was a retrospective review of patient records.

## Declaration of Competing Interest

This work was supported in part by MEXT KAKENHI grant number 15K08373 (NM) and AMED grant number 19ck0106323h003 (SIW).

HA has received unrestricted institutional funds from Eli Lilly Japan K.K., Pfizer Japan Inc., Shionogi & Co., Ltd., Ono Pharmaceutical Co., Ltd., Tsumura & Co., Taiho Pharmaceutical Co., Ltd., Meiji Seika Pharma Co., Ltd., Astellas Pharma Inc., and Covidien Japan Inc.

NM has received personal funds from AstraZeneca K. K., Chugai Pharmaceutical Co., Ltd., MSD K. K., Novartis Pharma K. K., Becton Dickinson and Company, and Covidien Japan Inc., and institutional research funding from Roche Diagnostics K. K., Ono Pharmaceutical Co., Ltd., and NEC Corporation, outside of this work.

## Appendix Supplementary materials

Image, application 1
