# Supplementary material for: Histological and prognostic data on surgically resected early-stage lung adenocarcinoma
Source: Data Brief. 2020 May 29;31:105785. doi: 10.1016/j.dib.2020.105785 (PMC7287229; doi:10.1016/j.dib.2020.105785)
Supplement: Supplementary file 1 [file mmc1.docx]

**Supplementary Table.** Data set of patient, operation, and histological information

| **No** | **Age** | **Gender** | **Smoking history** | **Operation** | **Right/Left** | **Lobe** | **Follow-up duration (year)** | **Survival status** | **Recurrence** | **Pathological total tumor diameter (cm)** | **Size of invasion (cm)** | **Histological subtype** | **INV-Category** | **ly** | **ｖ** |
| --- | --- | --- | --- | --- | --- | --- | --- | --- | --- | --- | --- | --- | --- | --- | --- |
| 1 | 26 | Male | Absent | Lob | Right | Upper | 1.0 | Dead | + | 2.6 | 2.6 | Papillary | INV-1 | 1 | 1 |
| 2 | 32 | Female | Absent | Lob | Right | Lower | 5.1 | Alive | - | 1.5 | 0.6 | Lepidic | INV-2 | 0 | 0 |
| 3 | 33 | Female | Absent | Seg | Right | Lower | 8.7 | Alive | - | 0.9 | 0.1 | Lepidic (MIA) | INV-1 | 0 | 0 |
| 4 | 33 | Male | Present | Wed | Left | Lower | 4.5 | Alive | - | 0.8 | 0.1 | Lepidic (MIA) | INV-2 | 0 | 0 |
| 5 | 34 | Female | Absent | Lob | Right | Upper | 9.1 | Alive | - | 1.0 | 1.0 | Acinar | INV-1 | 1 | 1 |
| 6 | 34 | Female | Present | Wed | Left | Lower | 6.5 | Alive | - | 0.8 | 0.0 | AIS | AIS | 0 | 0 |
| 7 | 36 | Male | Present | Lob | Left | Upper | 5.1 | Alive | - | 1.0 | 1.0 | Solid | INV-1 | 0 | 1 |
| 8 | 37 | Female | Absent | Seg | Left | Upper | 4.2 | Alive | - | 1.9 | 0.0 | AIS | AIS | 0 | 0 |
| 9 | 37 | Female | Absent | Seg | Right | Lower | 6.4 | Alive | - | 0.7 | 0.0 | AIS | AIS | 0 | 0 |
| 10 | 38 | Female | Present | Lob | Right | Middle | 4.0 | Alive | - | 1.8 | 0.0 | AIS | AIS | 0 | 0 |
| 11 | 39 | Female | Absent | Lob | Left | Upper | 8.8 | Alive | - | 2.2 | 0.2 | Lepidic (MIA) | INV-1 | 0 | 0 |
| 12 | 39 | Female | Absent | Seg | Right | Lower | 10.7 | Alive | - | 1.5 | 1.5 | Lepidic | INV-1 | 0 | 0 |
| 13 | 39 | Female | Present | Lob | Left | Upper | 5.6 | Alive | - | 1.3 | 0.9 | Lepidic | INV-1 | 0 | 0 |
| 14 | 39 | Male | Present | Seg | Right | Lower | 5.6 | Alive | - | 0.9 | 0.9 | Papillary | INV-1 | 0 | 0 |
| 15 | 40 | Female | Absent | Lob | Left | Lower | 5.0 | Alive | - | 1.2 | 0.0 | AIS | AIS | 0 | 0 |
| 16 | 40 | Female | Present | Lob | Right | Upper | 4.4 | Alive | - | 1.1 | 0.3 | Lepidic (MIA) | INV-2 | 0 | 0 |
| 17 | 41 | Female | Absent | Seg | Left | Upper | 5.5 | Alive | - | 1.0 | 0.6 | Papillary | INV-1 | 0 | 0 |
| 18 | 42 | Female | Present | Lob | Right | Upper | 7.1 | Alive | - | 1.5 | 0.7 | Lepidic | INV-2 | 0 | 0 |
| 19 | 42 | Male | Absent | Wed | Right | Upper | 9.0 | Alive | - | 1.1 | 0.0 | AIS | AIS | 0 | 0 |
| 20 | 42 | Male | Present | Lob | Right | Middle | 8.4 | Alive | - | 1.7 | 1.7 | Papillary | INV-1 | 0 | 0 |
| 21 | 43 | Female | Absent | Seg | Left | Upper | 5.5 | Alive | - | 1.2 | 1.0 | Papillary | INV-1 | 1 | 0 |
| 22 | 43 | Female | Present | Lob | Right | Middle | 4.4 | Alive | - | 1.2 | 1.2 | Acinar | INV-1 | 0 | 0 |
| 23 | 43 | Female | Present | Wed | Right | Upper | 4.1 | Alive | - | 1.4 | 0.0 | AIS | AIS | 0 | 0 |
| 24 | 43 | Male | Absent | Wed | Left | Upper | 6.1 | Alive | - | 0.5 | 0.0 | AIS | AIS | 0 | 0 |
| 25 | 43 | Male | Absent | Wed | Right | Upper | 6.1 | Alive | - | 1.2 | 0.0 | AIS | AIS | 0 | 0 |
| 26 | 44 | Female | Absent | Lob | Left | Upper | 7.8 | Alive | - | 2.6 | 1.0 | Papillary | INV-1 | 0 | 1 |
| 27 | 44 | Female | Absent | Wed | Left | Lower | 4.0 | Alive | - | 1.4 | 0.3 | Lepidic (MIA) | INV-2 | 0 | 0 |
| 28 | 44 | Male | Absent | Lob | Left | Upper | 4.1 | Alive | - | 2.0 | 0.1 | Lepidic (MIA) | INV-1 | 0 | 0 |
| 29 | 44 | Male | Present | Lob | Left | Upper | 6.5 | Alive | - | 1.9 | 0.7 | Lepidic | INV-1 | 0 | 0 |
| 30 | 45 | Female | Absent | Lob | Right | Lower | 8.2 | Alive | - | 1.8 | 0.6 | Lepidic | INV-2 | 0 | 0 |
| 31 | 45 | Female | Absent | Seg | Left | Upper | 6.0 | Alive | - | 1.8 | 0.0 | AIS | AIS | 0 | 0 |
| 32 | 45 | Female | Absent | Seg | Right | Upper | 5.6 | Alive | - | 1.1 | 0.0 | AIS | AIS | 0 | 0 |
| 33 | 45 | Female | Absent | Seg | Right | Upper | 4.5 | Alive | - | 1.7 | 0.2 | Lepidic (MIA) | INV-1 | 0 | 0 |
| 34 | 45 | Female | Present | Lob | Left | Lower | 4.7 | Alive | - | 2.2 | 2.2 | Papillary | INV-1 | 1 | 1 |
| 35 | 45 | Female | Present | Lob | Right | Lower | 6.6 | Alive | - | 2.1 | 0.4 | Lepidic (MIA) | INV-1 | 0 | 0 |
| 36 | 45 | Female | Present | Seg | Left | Upper | 5.2 | Alive | - | 0.8 | 0.0 | AIS | AIS | 0 | 0 |
| 37 | 45 | Male | Present | Lob | Left | Upper | 5.8 | Alive | - | 2.5 | 1.4 | Lepidic | INV-2 | 0 | 0 |
| 38 | 45 | Male | Present | Lob | Right | Upper | 5.8 | Alive | - | 1.3 | 0.4 | Lepidic (MIA) | INV-1 | 0 | 0 |
| 39 | 45 | Male | Present | Lob | Right | Upper | 5.8 | Alive | - | 0.9 | 0.3 | Lepidic (MIA) | INV-1 | 0 | 0 |
| 40 | 46 | Female | Absent | Lob | Left | Upper | 10.6 | Alive | - | 1.8 | 1.8 | Papillary | INV-1 | 1 | 0 |
| 41 | 46 | Female | Absent | Seg | Left | Upper | 6.1 | Alive | - | 1.0 | 0.0 | AIS | AIS | 0 | 0 |
| 42 | 46 | Female | Absent | Wed | Left | Upper | 5.2 | Alive | - | 0.7 | 0.0 | AIS | AIS | 0 | 0 |
| 43 | 46 | Male | Absent | Wed | Right | Upper | 5.3 | Alive | - | 0.8 | 0.0 | AIS | AIS | 0 | 0 |
| 44 | 46 | Male | Present | Lob | Right | Upper | 6.3 | Alive | - | 1.7 | 0.7 | Lepidic | INV-2 | 0 | 0 |
| 45 | 46 | Male | Present | Seg | Right | Lower | 6.1 | Alive | - | 1.5 | 0.2 | Lepidic (MIA) | INV-2 | 0 | 0 |
| 46 | 47 | Female | Absent | Lob | Left | Upper | 4.1 | Alive | + | 1.4 | 0.7 | Papillary | INV-1 | 0 | 0 |
| 47 | 47 | Female | Absent | Lob | Right | Middle | 2.5 | Dead | - | 1.5 | 0.3 | Lepidic (MIA) | INV-1 | 0 | 0 |
| 48 | 47 | Female | Absent | Lob | Right | Middle | 4.9 | Alive | - | 1.0 | 1.0 | Micropapillary | INV-1 | 1 | 0 |
| 49 | 47 | Female | Absent | Lob | Right | Upper | 5.2 | Alive | - | 2.0 | 0.9 | Papillary | INV-1 | 0 | 0 |
| 50 | 47 | Female | Absent | Seg | Left | Lower | 4.5 | Alive | - | 1.2 | 1.3 | Lepidic | INV-2 | 0 | 0 |
| 51 | 47 | Female | Present | Lob | Right | Upper | 8.0 | Alive | - | 2.2 | 0.0 | AIS | AIS | 0 | 0 |
| 52 | 47 | Male | Present | Lob | Right | Upper | 3.7 | Dead | - | 2.9 | 2.9 | Micropapillary | INV-1 | 0 | 0 |
| 53 | 47 | Male | Present | Seg | Right | Lower | 5.5 | Alive | - | 0.9 | 0.9 | Papillary | INV-1 | 0 | 0 |
| 54 | 48 | Female | Absent | Bil | Right | Lower | 4.2 | Alive | - | 2.8 | 0.6 | Lepidic | INV-1 | 0 | 0 |
| 55 | 48 | Female | Absent | Lob | Right | Middle | 6.8 | Alive | - | 1.3 | 0.5 | Lepidic (MIA) | INV-1 | 0 | 1 |
| 56 | 48 | Female | Present | Wed | Left | Upper | 4.5 | Alive | - | 1.2 | 0.0 | AIS | AIS | 0 | 0 |
| 57 | 48 | Male | Absent | Lob | Left | Lower | 9.7 | Alive | - | 1.3 | 0.2 | Lepidic (MIA) | INV-1 | 0 | 0 |
| 58 | 48 | Male | Absent | Wed | Right | Upper | 6.9 | Alive | - | 0.8 | 0.0 | AIS | AIS | 0 | 0 |
| 59 | 49 | Female | Absent | Lob | Right | Middle | 5.5 | Alive | - | 1.8 | 1.2 | Lepidic | INV-1 | 0 | 0 |
| 60 | 49 | Female | Absent | Wed | Left | Upper | 7.6 | Alive | - | 0.8 | 0.0 | AIS | AIS | 0 | 0 |
| 61 | 49 | Female | Absent | Wed | Left | Upper | 3.8 | Alive | + | 1.5 | 1.0 | Lepidic | INV-1 | 0 | 0 |
| 62 | 49 | Female | Absent | Wed | Right | Lower | 5.0 | Alive | - | 0.9 | 0.8 | Papillary | INV-1 | 0 | 0 |
| 63 | 49 | Female | Present | Lob | Right | Lower | 5.8 | Alive | - | 1.4 | 1.4 | Papillary | INV-1 | 1 | 1 |
| 64 | 49 | Female |  | Lob | Left | Upper | 8.0 | Alive | - | 2.5 | 0.9 | Lepidic | INV-1 | 0 | 0 |
| 65 | 49 | Male | Absent | Lob | Right | Upper | 9.5 | Alive | - | 2.5 | 0.4 | Lepidic (MIA) | INV-1 | 0 | 0 |
| 66 | 49 | Male | Absent | Lob | Right | Upper | 5.5 | Alive | - | 2.2 | 0.7 | Lepidic | INV-1 | 0 | 0 |
| 67 | 49 | Male | Absent | Wed | Left | Lower | 6.2 | Alive | - | 0.7 | 0.0 | AIS | AIS | 0 | 0 |
| 68 | 49 | Male | Present | Lob | Right | Middle | 5.6 | Alive | - | 2.3 | 2.3 | Papillary | INV-1 | 0 | 0 |
| 69 | 49 | Male | Present | Lob | Right | Middle | 4.9 | Alive | - | 1.1 | 0.0 | AIS | AIS | 0 | 0 |
| 70 | 49 | Male | Present | Seg | Left | Upper | 6.6 | Alive | - | 2.2 | 0.4 | Lepidic (MIA) | INV-1 | 0 | 0 |
| 71 | 49 | Male | Present | Seg | Right | Lower | 6.0 | Alive | - | 1.0 | 0.0 | AIS | AIS | 0 | 0 |
| 72 | 50 | Female | Absent | Lob | Left | Upper | 3.7 | Alive | - | 1.9 | 0.6 | Lepidic | INV-2 | 0 | 0 |
| 73 | 50 | Female | Present | Lob | Left | Lower | 8.0 | Alive | - | 1.3 | 0.8 | Acinar | INV-1 | 0 | 0 |
| 74 | 50 | Female | Present | Lob | Right | Lower | 8.8 | Alive | - | 1.2 | 0.7 | Lepidic | INV-2 | 0 | 0 |
| 75 | 50 | Female | Present | Lob | Right | Upper | 5.5 | Alive | - | 2.2 | 1.0 | Lepidic | INV-2 | 0 | 0 |
| 76 | 50 | Female | Present | Wed | Left | Lower | 5.0 | Alive | - | 1.6 | 0.8 | Acinar | INV-1 | 0 | 0 |
| 77 | 50 | Female | Present | Wed | Right | Upper | 5.6 | Alive | - | 0.9 | 0.4 | Lepidic (MIA) | INV-2 | 0 | 0 |
| 78 | 50 | Male | Absent | Lob | Right | Lower | 9.8 | Alive | - | 1.4 | 1.4 | Lepidic | INV-1 | 0 | 0 |
| 79 | 50 | Male | Present | Lob | Right | Upper | 1.3 | Alive | + | 2.3 | 2.3 | Papillary | INV-1 | 0 | 1 |
| 80 | 51 | Female | Absent | Lob | Left | Upper | 8.2 | Alive | - | 1.9 | 0.5 | Lepidic (MIA) | INV-2 | 0 | 0 |
| 81 | 51 | Female | Absent | Lob | Right | Lower | 6.5 | Alive | - | 1.8 | 0.1 | Lepidic (MIA) | INV-1 | 0 | 0 |
| 82 | 51 | Female | Absent | Lob | Right | Upper | 2.9 | Alive | - | 0.9 | 0.0 | AIS | AIS | 0 | 0 |
| 83 | 51 | Female | Absent | Seg | Left | Lower | 4.9 | Alive | - | 1.3 | 0.1 | Lepidic (MIA) | INV-2 | 0 | 0 |
| 84 | 51 | Female | Absent | Seg | Left | Upper | 5.7 | Alive | - | 1.2 | 0.5 | Lepidic (MIA) | INV-1 | 0 | 0 |
| 85 | 51 | Female | Present | Lob | Right | Upper | 4.7 | Alive | - | 2.0 | 2.0 | Papillary | INV-1 | 0 | 1 |
| 86 | 51 | Female | Present | Seg | Left | Lower | 6.1 | Alive | - | 1.0 | 0.2 | Lepidic (MIA) | INV-1 | 0 | 0 |
| 87 | 51 | Male | Absent | Lob | Left | Upper | 8.1 | Alive | - | 1.5 | 1.0 | Lepidic | INV-1 | 0 | 0 |
| 88 | 51 | Male | Absent | Lob | Left | Upper | 3.9 | Alive | - | 1.5 | 1.5 | Acinar | INV-1 | 0 | 0 |
| 89 | 51 | Male | Absent | Seg | Left | Lower | 4.7 | Alive | - | 1.2 | 0.2 | Lepidic (MIA) | INV-1 | 0 | 0 |
| 90 | 51 | Male | Present | Lob | Left | Lower | 6.0 | Alive | - | 2.2 | 2.2 | Papillary | INV-1 | 0 | 1 |
| 91 | 51 | Male | Present | Lob | Left | Upper | 7.0 | Alive | - | 3.0 | 1.2 | Papillary | INV-1 | 0 | 0 |
| 92 | 51 | Male | Present | Seg | Right | Lower | 5.5 | Alive | - | 2.8 | 1.2 | Lepidic | INV-2 | 0 | 0 |
| 93 | 52 | Female | Absent | Lob | Left | Lower | 8.0 | Alive | - | 1.2 | 0.2 | Lepidic (MIA) | INV-1 | 0 | 0 |
| 94 | 52 | Female | Absent | Lob | Right | Lower | 5.4 | Alive | - | 2.9 | 0.1 | Lepidic (MIA) | INV-1 | 0 | 0 |
| 95 | 52 | Female | Absent | Lob | Right | Middle | 2.0 | Alive | + | 2.5 | 0.2 | Lepidic (MIA) | INV-1 | 0 | 0 |
| 96 | 52 | Female | Absent | Lob | Right | Upper | 5.2 | Alive | - | 3.0 | 3.0 | Lepidic | INV-1 | 0 | 0 |
| 97 | 52 | Female | Absent | Lob | Right | Upper | 5.5 | Alive | - | 1.9 | 0.4 | Lepidic (MIA) | INV-1 | 0 | 0 |
| 98 | 52 | Female | Absent | Seg | Left | Upper | 6.4 | Alive | - | 1.2 | 0.0 | AIS | AIS | 0 | 0 |
| 99 | 52 | Female | Present | Lob | Left | Upper | 5.3 | Alive | - | 2.6 | 2.6 | Papillary | INV-1 | 0 | 0 |
| 100 | 52 | Female | Present | Lob | Right | Lower | 8.7 | Alive | - | 1.9 | 0.4 | Lepidic (MIA) | INV-1 | 1 | 0 |
| 101 | 52 | Female | Present | Lob | Right | Upper | 5.1 | Alive | - | 1.7 | 1.7 | Papillary | INV-1 | 0 | 1 |
| 102 | 52 | Female | Present | Wed | Left | Lower | 3.3 | Alive | + | 2.1 | 2.1 | Papillary | INV-1 | 1 | 0 |
| 103 | 52 | Female | Present | Wed | Right | Lower | 4.1 | Alive | - | 1.1 | 0.6 | Lepidic | INV-2 | 0 | 0 |
| 104 | 52 | Male | Absent | Lob | Right | Upper | 5.4 | Alive | - | 2.5 | 0.3 | Lepidic (MIA) | INV-2 | 0 | 0 |
| 105 | 52 | Male | Absent | Seg | Right | Lower | 5.2 | Alive | - | 0.8 | 0.0 | AIS | AIS | 0 | 0 |
| 106 | 52 | Male | Absent | Wed | Right | Upper | 5.0 | Alive | - | 1.5 | 0.4 | Lepidic (MIA) | INV-2 | 0 | 0 |
| 107 | 52 | Male | Present | Lob | Right | Lower | 5.1 | Alive | - | 1.5 | 1.5 | Micropapillary | INV-1 | 0 | 0 |
| 108 | 53 | Female | Absent | Lob | Left | Lower | 6.4 | Alive | - | 1.4 | 1.4 | Acinar | INV-1 | 0 | 0 |
| 109 | 53 | Female | Absent | Lob | Left | Upper | 5.8 | Alive | - | 1.7 | 1.7 | Lepidic | INV-1 | 0 | 0 |
| 110 | 53 | Female | Absent | Lob | Left | Upper | 6.1 | Alive | - | 1.7 | 0.3 | Lepidic (MIA) | INV-1 | 0 | 0 |
| 111 | 53 | Female | Absent | Lob | Right | Middle | 8.6 | Alive | - | 1.4 | 1.2 | Acinar | INV-1 | 0 | 0 |
| 112 | 53 | Female | Absent | Lob | Right | Middle | 5.4 | Alive | - | 1.5 | 0.8 | Lepidic | INV-1 | 0 | 0 |
| 113 | 53 | Female | Absent | Wed | Right | Lower | 2.8 | Alive | - | 0.5 | 0.0 | AIS | AIS | 0 | 0 |
| 114 | 53 | Female | Absent | Wed | Right | Lower | 4.9 | Alive | - | 1.1 | 1.0 | Lepidic (MIA) | INV-2 | 0 | 0 |
| 115 | 53 | Female | Present | Lob | Right | Upper | 5.9 | Alive | - | 2.3 | 0.2 | Lepidic (MIA) | INV-1 | 0 | 0 |
| 116 | 53 | Male | Absent | Lob | Left | Upper | 5.7 | Alive | - | 1.6 | 1.0 | Lepidic | INV-2 | 0 | 0 |
| 117 | 53 | Male | Absent | Lob | Right | Lower | 8.4 | Alive | - | 2.5 | 2.5 | Lepidic | INV-1 | 1 | 0 |
| 118 | 53 | Male | Present | Seg | Left | Upper | 5.5 | Alive | - | 2.2 | 2.2 | Micropapillary | INV-1 | 1 | 1 |
| 119 | 53 | Male | Present | Seg | Right | Lower | 8.4 | Alive | - | 2.0 | 1.4 | Lepidic | INV-1 | 0 | 0 |
| 120 | 53 | Male | Present | Seg | Right | Lower | 2.6 | Alive | - | 1.3 | 0.1 | Lepidic (MIA) | INV-2 | 0 | 0 |
| 121 | 54 | Female | Absent | Lob | Left | Lower | 5.3 | Alive | - | 1.5 | 1.3 | Lepidic | INV-1 | 0 | 0 |
| 122 | 54 | Female | Absent | Lob | Left | Lower | 5.8 | Alive | - | 1.8 | 0.5 | Lepidic (MIA) | INV-1 | 0 | 0 |
| 123 | 54 | Female | Absent | Lob | Left | Upper | 5.9 | Alive | - | 1.9 | 1.9 | Solid | INV-1 | 1 | 1 |
| 124 | 54 | Female | Absent | Lob | Right | Upper | 9.2 | Alive | - | 1.7 | 0.7 | Lepidic | INV-2 | 0 | 0 |
| 125 | 54 | Female | Absent | Seg | Right | Upper | 2.1 | Alive | - | 1.8 | 0.3 | Lepidic (MIA) | INV-1 | 0 | 0 |
| 126 | 54 | Female | Absent | Wed | Left | Upper | 7.4 | Alive | - | 1.6 | 0.0 | AIS | AIS | 0 | 0 |
| 127 | 54 | Female | Absent | Wed | Right | Lower | 7.9 | Alive | - | 1.2 | 0.0 | AIS | AIS | 0 | 0 |
| 128 | 54 | Female | Present | Lob | Left | Upper | 0.4 | Alive | + | 1.8 | 1.8 | Solid | INV-1 | 0 | 1 |
| 129 | 54 | Female | Present | Lob | Right | Lower | 9.4 | Alive | - | 2.1 | 2.1 | Papillary | INV-1 | 1 | 1 |
| 130 | 54 | Female | Present | Lob | Right | Upper | 1.2 | Alive | - | 2.5 | 0.5 | Lepidic (MIA) | INV-1 | 0 | 0 |
| 131 | 54 | Female | Present | Wed | Right | Upper | 4.3 | Alive | - | 0.8 | 0.0 | AIS | AIS | 0 | 0 |
| 132 | 54 | Female | Present | Wed | Right | Upper | 4.6 | Alive | - | 0.7 | 0.7 | Acinar | INV-1 | 1 | 1 |
| 133 | 54 | Male | Absent | Lob | Right | Lower | 5.7 | Alive | - | 2.0 | 0.8 | Lepidic | INV-2 | 0 | 0 |
| 134 | 54 | Male | Absent | Lob | Right | Upper | 4.8 | Alive | - | 2.0 | 2.0 | Papillary | INV-1 | 1 | 1 |
| 135 | 54 | Male | Present | Lob | Right | Lower | 3.0 | Dead | + | 2.4 | 2.4 | Solid | INV-1 | 0 | 0 |
| 136 | 54 | Male | Present | Lob | Right | Lower | 5.2 | Alive | - | 1.5 | 1.5 | Papillary | INV-1 | 0 | 0 |
| 137 | 54 | Male | Present | Lob | Right | Upper | 2.0 | Alive | + | 1.3 | 0.7 | Lepidic | INV-1 | 0 | 0 |
| 138 | 54 | Male | Present | Seg | Right | Lower | 3.9 | Alive | - | 1.3 | 1.3 | Papillary | INV-1 | 0 | 0 |
| 139 | 55 | Female | Absent | Lob | Left | Upper | 4.6 | Alive | - | 3.0 | 1.2 | Lepidic | INV-1 | 0 | 0 |
| 140 | 55 | Female | Absent | Lob | Right | Lower | 7.3 | Alive | - | 1.4 | 0.8 | Papillary | INV-1 | 0 | 0 |
| 141 | 55 | Female | Absent | Lob | Right | Lower | 9.2 | Alive | - | 1.6 | 1.3 | Solid | INV-1 | 0 | 0 |
| 142 | 55 | Female | Absent | Lob | Right | Lower | 5.8 | Alive | - | 2.6 | 2.6 | Papillary | INV-1 | 0 | 0 |
| 143 | 55 | Female | Absent | Lob | Right | Upper | 8.6 | Alive | - | 2.8 | 2.8 | Lepidic | INV-1 | 1 | 0 |
| 144 | 55 | Female | Absent | Lob | Right | Upper | 7.5 | Alive | - | 1.4 | 0.9 | Lepidic | INV-2 | 0 | 0 |
| 145 | 55 | Female | Absent | Seg | Left | Lower | 5.1 | Alive | - | 0.8 | 0.0 | AIS | AIS | 0 | 0 |
| 146 | 55 | Female | Absent | Seg | Left | Upper | 8.4 | Alive | - | 1.9 | 0.0 | AIS | AIS | 0 | 0 |
| 147 | 55 | Female | Absent | Seg | Right | Lower | 7.9 | Alive | - | 1.7 | 1.3 | Lepidic | INV-1 | 0 | 0 |
| 148 | 55 | Female | Absent | Wed | Left | Lower | 5.7 | Alive | - | 1.3 | 0.3 | Lepidic (MIA) | INV-2 | 0 | 0 |
| 149 | 55 | Female | Absent | Wed | Right | Lower | 5.6 | Alive | - | 1.3 | 0.4 | Lepidic (MIA) | INV-1 | 0 | 0 |
| 150 | 55 | Female | Absent | Wed | Right | Upper | 4.6 | Alive | - | 1.2 | 0.4 | Lepidic (MIA) | INV-2 | 0 | 0 |
| 151 | 55 | Female | Absent | Wed | Right | Upper | 5.7 | Alive | - | 1.0 | 0.0 | AIS | AIS | 0 | 0 |
| 152 | 55 | Female | Present | Lob | Right | Lower | 4.5 | Alive | - | 2.7 | 2.1 | Papillary | INV-1 | 0 | 0 |
| 153 | 55 | Female | Present | Lob | Right | Lower | 5.3 | Alive | - | 0.9 | 0.9 | Papillary | INV-1 | 0 | 0 |
| 154 | 55 | Female | Present | Lob | Right | Lower | 5.5 | Alive | - | 2.1 | 2.1 | Papillary | INV-1 | 0 | 0 |
| 155 | 55 | Female | Present | Lob | Right | Upper | 6.2 | Alive | - | 1.2 | 0.1 | Lepidic (MIA) | INV-2 | 0 | 0 |
| 156 | 55 | Female | Present | Wed | Right | Upper | 6.4 | Alive | - | 1.2 | 0.0 | AIS | AIS | 0 | 0 |
| 157 | 55 | Female | Present | Wed | Right | Upper | 6.4 | Alive | - | 1.0 | 0.0 | AIS | AIS | 0 | 0 |
| 158 | 55 | Male | Absent | Lob | Left | Upper | 6.3 | Alive | - | 0.7 | 0.4 | Lepidic (MIA) | INV-1 | 0 | 0 |
| 159 | 55 | Male | Absent | Lob | Left | Upper | 5.7 | Alive | - | 1.2 | 0.7 | Lepidic | INV-1 | 0 | 0 |
| 160 | 55 | Male | Absent | Lob | Right | Lower | 6.0 | Alive | - | 2.0 | 1.8 | Papillary | INV-1 | 0 | 0 |
| 161 | 55 | Male | Absent | Lob | Right | Upper | 5.0 | Alive | - | 1.2 | 1.2 | Lepidic | INV-1 | 0 | 0 |
| 162 | 55 | Male | Absent | Wed | Left | Upper | 6.0 | Alive | - | 1.4 | 0.0 | AIS | AIS | 0 | 0 |
| 163 | 55 | Male | Present | Lob | Left | Lower | 2.5 | Alive | - | 1.5 | 0.2 | Lepidic (MIA) | INV-2 | 0 | 0 |
| 164 | 55 | Male | Present | Lob | Left | Upper | 1.8 | Alive | + | 1.5 | 1.5 | Acinar | INV-1 | 0 | 0 |
| 165 | 55 | Male | Present | Lob | Left | Upper | 8.1 | Alive | - | 2.6 | 1.6 | Papillary | INV-1 | 0 | 0 |
| 166 | 55 | Male | Present | Lob | Left | Upper | 1.4 | Alive | + | 2.5 | 2.5 | Solid | INV-1 | 0 | 1 |
| 167 | 55 | Male | Present | Lob | Right | Lower | 5.6 | Alive | - | 2.4 | 2.0 | Lepidic | INV-1 | 0 | 0 |
| 168 | 55 | Male | Present | Lob | Right | Upper | 6.1 | Alive | - | 1.7 | 1.7 | Lepidic | INV-1 | 0 | 0 |
| 169 | 55 | Male | Present | Lob | Right | Upper | 6.5 | Alive | - | 1.8 | 1.0 | Papillary | INV-1 | 0 | 1 |
| 170 | 55 | Male | Present | Seg | Left | Lower | 5.6 | Alive | - | 1.0 | 1.0 | Lepidic | INV-1 | 0 | 0 |
| 171 | 55 | Male | Present | Wed | Left | Upper | 2.0 | Alive | + | 1.5 | 0.6 | Papillary | INV-1 | 0 | 0 |
| 172 | 55 | Male | Present | Wed | Right | Upper | 5.0 | Alive | - | 2.2 | 0.1 | Lepidic (MIA) | INV-2 | 0 | 0 |
| 173 | 56 | Female | Absent | Lob | Left | Lower | 8.4 | Alive | - | 2.9 | 0.8 | Papillary | INV-1 | 1 | 1 |
| 174 | 56 | Female | Absent | Lob | Right | Upper | 7.2 | Alive | - | 2.8 | 0.0 | AIS | AIS | 0 | 0 |
| 175 | 56 | Female | Absent | Lob | Right | Upper | 7.6 | Alive | - | 1.8 | 0.2 | Lepidic (MIA) | INV-1 | 0 | 1 |
| 176 | 56 | Female | Absent | Lob | Right | Upper | 6.6 | Alive | - | 1.6 | 0.2 | Lepidic (MIA) | INV-1 | 0 | 0 |
| 177 | 56 | Female | Absent | Lob | Right | Upper | 6.0 | Alive | - | 1.8 | 1.8 | Acinar | INV-1 | 0 | 0 |
| 178 | 56 | Female | Absent | Lob | Right | Upper | 5.6 | Alive | - | 2.1 | 0.4 | Lepidic (MIA) | INV-1 | 0 | 0 |
| 179 | 56 | Female | Absent | Seg | Left | Upper | 1.7 | Dead | + | 0.7 | 0.2 | Lepidic (MIA) | INV-1 | 0 | 0 |
| 180 | 56 | Female | Absent | Seg | Right | Lower | 5.1 | Alive | - | 2.4 | 0.3 | Lepidic (MIA) | INV-1 | 0 | 0 |
| 181 | 56 | Female | Absent | Wed | Left | Upper | 5.1 | Alive | - | 0.8 | 0.0 | AIS | AIS | 0 | 0 |
| 182 | 56 | Female | Present | Lob | Right | Upper | 4.5 | Alive | - | 2.1 | 1.8 | Lepidic | INV-1 | 0 | 0 |
| 183 | 56 | Female | Present | Lob | Right | Upper | 4.2 | Alive | - | 2.7 | 2.7 | Papillary | INV-1 | 1 | 0 |
| 184 | 56 | Female | Present | Seg | Right | Upper | 5.3 | Alive | - | 1.5 | 0.3 | Lepidic (MIA) | INV-1 | 0 | 0 |
| 185 | 56 | Male | Absent | Lob | Left | Lower | 5.7 | Alive | - | 2.3 | 0.5 | Lepidic (MIA) | INV-2 | 0 | 0 |
| 186 | 56 | Male | Absent | Lob | Right | Lower | 4.5 | Alive | - | 0.5 | 0.5 | Lepidic | INV-1 | 0 | 0 |
| 187 | 56 | Male | Present | Lob | Left | Upper | 5.3 | Alive | - | 2.3 | 2.3 | Papillary | INV-1 | 0 | 1 |
| 188 | 56 | Male | Present | Lob | Right | Upper | 4.7 | Alive | - | 1.5 | 1.5 | Lepidic | INV-1 | 0 | 0 |
| 189 | 56 | Male | Present | Lob | Right | Upper | 8.6 | Alive | - | 2.2 | 0.2 | Lepidic (MIA) | INV-1 | 0 | 0 |
| 190 | 56 | Male | Present | Lob | Right | Upper | 4.9 | Alive | - | 1.6 | 1.6 | Acinar | INV-1 | 0 | 0 |
| 191 | 56 | Male | Present | Lob | Right | Upper | 4.0 | Alive | + | 1.9 | 1.9 | Acinar | INV-1 | 1 | 0 |
| 192 | 56 | Male | Present | Wed | Left | Upper | 5.5 | Alive | - | 1.5 | 0.0 | AIS | AIS | 0 | 0 |
| 193 | 57 | Female | Absent | Lob | Left | Lower | 5.6 | Alive | - | 2.1 | 0.9 | Papillary | INV-1 | 0 | 0 |
| 194 | 57 | Female | Absent | Lob | Left | Upper | 7.3 | Alive | - | 1.8 | 0.8 | Lepidic | INV-1 | 0 | 0 |
| 195 | 57 | Female | Absent | Lob | Left | Upper | 8.7 | Alive | - | 1.4 | 0.2 | Lepidic (MIA) | INV-1 | 1 | 0 |
| 196 | 57 | Female | Absent | Lob | Right | Lower | 6.3 | Alive | - | 1.5 | 0.7 | Papillary | INV-1 | 1 | 1 |
| 197 | 57 | Female | Absent | Lob | Right | Lower | 6.8 | Alive | - | 1.9 | 0.4 | Lepidic (MIA) | INV-1 | 0 | 0 |
| 198 | 57 | Female | Absent | Lob | Right | Upper | 8.6 | Alive | - | 2.2 | 1.4 | Lepidic | INV-2 | 0 | 0 |
| 199 | 57 | Female | Absent | Lob | Right | Upper | 4.3 | Alive | - | 1.2 | 1.2 | Papillary | INV-1 | 0 | 0 |
| 200 | 57 | Female | Absent | Lob | Right | Upper | 1.5 | Alive | - | 2.1 | 0.1 | Lepidic (MIA) | INV-2 | 0 | 0 |
| 201 | 57 | Female | Absent | Seg | Left | Lower | 8.2 | Alive | - | 1.0 | 1.0 | Lepidic | INV-2 | 0 | 0 |
| 202 | 57 | Female | Present | Lob | Left | Lower | 1.7 | Alive | - | 1.9 | 1.9 | Lepidic | INV-1 | 1 | 1 |
| 203 | 57 | Female | Present | Lob | Left | Upper | 4.6 | Alive | - | 1.7 | 1.7 | Papillary | INV-1 | 0 | 1 |
| 204 | 57 | Female | Present | Lob | Right | Upper | 4.0 | Alive | - | 0.9 | 0.9 | Papillary | INV-1 | 0 | 0 |
| 205 | 57 | Female | Present | Seg | Left | Upper | 5.1 | Alive | - | 1.4 | 0.5 | Lepidic (MIA) | INV-1 | 0 | 0 |
| 206 | 57 | Male | Absent | Lob | Left | Lower | 5.2 | Alive | - | 2.1 | 0.8 | Lepidic | INV-1 | 0 | 0 |
| 207 | 57 | Male | Absent | Lob | Right | Upper | 3.2 | Alive | + | 2.5 | 2.5 | Papillary | INV-1 | 1 | 1 |
| 208 | 57 | Male | Absent | Seg | Left | Lower | 8.3 | Alive | - | 1.5 | 0.8 | Lepidic | INV-1 | 0 | 0 |
| 209 | 57 | Male | Absent | Seg | Right | Lower | 7.3 | Alive | - | 1.2 | 0.6 | Lepidic | INV-1 | 0 | 0 |
| 210 | 57 | Male | Present | Lob | Left | Upper | 6.2 | Alive | - | 1.9 | 1.9 | Solid | INV-1 | 0 | 1 |
| 211 | 57 | Male | Present | Lob | Right | Lower | 8.2 | Alive | - | 1.9 | 1.9 | Acinar | INV-1 | 1 | 0 |
| 212 | 57 | Male | Present | Lob | Right | Upper | 4.6 | Alive | - | 3.0 | 3.0 | Lepidic | INV-1 | 0 | 0 |
| 213 | 57 | Male | Present | Seg | Left | Upper | 4.4 | Alive | - | 0.9 | 0.1 | Lepidic (MIA) | INV-2 | 0 | 0 |
| 214 | 57 | Male | Present | Seg | Left | Upper | 4.2 | Alive | - | 1.0 | 0.0 | AIS | AIS | 0 | 0 |
| 215 | 57 | Male | Present | Seg | Left | Upper | 2.7 | Alive | - | 1.1 | 0.5 | Lepidic (MIA) | INV-1 | 0 | 0 |
| 216 | 57 | Male | Present | Wed | Right | Lower | 4.1 | Alive | - | 0.6 | 0.6 | Lepidic | INV-1 | 0 | 0 |
| 217 | 57 | Male |  | Lob | Left | Upper | 5.1 | Alive | - | 1.7 | 0.2 | Lepidic (MIA) | INV-2 | 0 | 0 |
| 218 | 58 | Female | Absent | Lob | Left | Upper | 7.9 | Alive | - | 1.8 | 1.8 | Solid | INV-1 | 0 | 1 |
| 219 | 58 | Female | Absent | Lob | Left | Upper | 5.2 | Alive | - | 2.5 | 2.5 | Papillary | INV-1 | 0 | 0 |
| 220 | 58 | Female | Absent | Lob | Right | Lower | 5.4 | Alive | - | 1.7 | 0.2 | Lepidic (MIA) | INV-1 | 0 | 1 |
| 221 | 58 | Female | Absent | Lob | Right | Upper | 4.8 | Alive | - | 1.1 | 0.4 | Lepidic (MIA) | INV-1 | 0 | 0 |
| 222 | 58 | Female | Absent | Lob | Right | Upper | 10.1 | Alive | - | 3.0 | 1.8 | Lepidic | INV-2 | 0 | 0 |
| 223 | 58 | Female | Absent | Lob | Right | Upper | 7.0 | Alive | - | 2.5 | 1.6 | Solid | INV-1 | 0 | 1 |
| 224 | 58 | Female | Absent | Lob | Right | Upper | 2.1 | Alive | - | 2.0 | 2.0 | Lepidic | INV-1 | 0 | 0 |
| 225 | 58 | Female | Absent | Lob | Right | Upper | 5.6 | Alive | - | 1.8 | 1.0 | Papillary | INV-1 | 1 | 1 |
| 226 | 58 | Female | Absent | Seg | Left | Upper | 6.1 | Alive | - | 1.5 | 0.5 | Lepidic (MIA) | INV-2 | 0 | 0 |
| 227 | 58 | Female | Absent | Seg | Right | Lower | 5.3 | Alive | - | 1.8 | 0.8 | Lepidic | INV-2 | 0 | 0 |
| 228 | 58 | Female | Absent | Seg | Right | Lower | 6.3 | Alive | - | 0.9 | 0.0 | AIS | AIS | 0 | 0 |
| 229 | 58 | Female | Absent | Wed | Left | Lower | 2.0 | Alive | - | 1.0 | 0.0 | AIS | AIS | 0 | 0 |
| 230 | 58 | Female | Absent | Wed | Right | Lower | 4.4 | Alive | - | 0.7 | 0.1 | Lepidic (MIA) | INV-1 | 0 | 0 |
| 231 | 58 | Female | Absent | Wed | Right | Lower | 3.1 | Alive | - | 1.0 | 0.0 | AIS | AIS | 0 | 0 |
| 232 | 58 | Female | Present | Lob | Left | Lower | 8.2 | Alive | - | 1.5 | 1.2 | Papillary | INV-1 | 0 | 0 |
| 233 | 58 | Female | Present | Lob | Left | Lower | 1.4 | Alive | - | 2.6 | 2.6 | Papillary | INV-1 | 0 | 0 |
| 234 | 58 | Female | Present | Lob | Left | Upper | 11.3 | Alive | - | 1.8 | 1.8 | Lepidic | INV-1 | 0 | 0 |
| 235 | 58 | Female | Present | Lob | Left | Upper | 5.7 | Alive | - | 2.8 | 2.8 | Papillary | INV-1 | 1 | 1 |
| 236 | 58 | Female | Present | Lob | Left | Upper | 6.7 | Alive | - | 2.4 | 0.5 | Lepidic (MIA) | INV-1 | 0 | 0 |
| 237 | 58 | Female | Present | Seg | Left | Lower | 8.5 | Alive | - | 1.0 | 0.3 | Lepidic (MIA) | INV-2 | 0 | 0 |
| 238 | 58 | Female | Present | Seg | Right | Lower | 3.5 | Alive | - | 0.8 | 0.0 | AIS | AIS | 0 | 0 |
| 239 | 58 | Female | Present | Seg | Right | Lower | 4.4 | Alive | - | 1.4 | 0.8 | Papillary | INV-1 | 0 | 0 |
| 240 | 58 | Female | Present | Wed | Left | Lower | 4.8 | Alive | - | 1.5 | 0.0 | AIS | AIS | 0 | 0 |
| 241 | 58 | Female |  | Lob | Right | Middle | 6.5 | Alive | - | 1.8 | 1.2 | Lepidic | INV-1 | 0 | 0 |
| 242 | 58 | Male | Absent | Lob | Right | Upper | 5.8 | Alive | - | 2.5 | 0.6 | Lepidic | INV-1 | 0 | 0 |
| 243 | 58 | Male | Present | Lob | Left | Lower | 4.0 | Alive | - | 1.5 | 0.6 | Lepidic | INV-1 | 0 | 0 |
| 244 | 58 | Male | Present | Lob | Right | Lower | 5.9 | Alive | - | 2.7 | 2.7 | Papillary | INV-1 | 1 | 0 |
| 245 | 58 | Male | Present | Lob | Right | Upper | 7.1 | Alive | - | 0.9 | 0.9 | Solid | INV-1 | 0 | 0 |
| 246 | 58 | Male | Present | Lob | Right | Upper | 5.5 | Alive | - | 2.8 | 1.2 | Lepidic | INV-1 | 0 | 0 |
| 247 | 58 | Male | Present | Lob | Right | Upper | 6.3 | Alive | - | 1.7 | 1.7 | Solid | INV-1 | 0 | 0 |
| 248 | 58 | Male | Present | Lob | Right | Upper | 4.6 | Alive | - | 2.2 | 1.9 | Papillary | INV-1 | 0 | 0 |
| 249 | 58 | Male | Present | Seg | Left | Upper | 1.4 | Dead | - | 2.0 | 0.2 | Lepidic (MIA) | INV-1 | 0 | 0 |
| 250 | 58 | Male | Present | Seg | Left | Upper | 6.4 | Alive | - | 1.1 | 0.0 | AIS | AIS | 0 | 0 |
| 251 | 58 | Male | Present | Wed | Right | Lower | 4.5 | Alive | - | 2.0 | 0.7 | Lepidic | INV-2 | 0 | 0 |
| 252 | 59 | Female | Absent | Lob | Left | Lower | 6.9 | Alive | - | 1.2 | 0.8 | Lepidic | INV-1 | 0 | 0 |
| 253 | 59 | Female | Absent | Lob | Left | Lower | 5.6 | Alive | - | 2.7 | 2.7 | Papillary | INV-1 | 1 | 0 |
| 254 | 59 | Female | Absent | Lob | Left | Upper | 9.1 | Alive | - | 2.3 | 1.4 | Lepidic | INV-2 | 0 | 0 |
| 255 | 59 | Female | Absent | Lob | Left | Upper | 5.4 | Alive | - | 1.6 | 1.4 | Lepidic | INV-1 | 0 | 0 |
| 256 | 59 | Female | Absent | Lob | Right | Lower | 6.0 | Alive | - | 1.2 | 0.2 | Lepidic (MIA) | INV-1 | 0 | 0 |
| 257 | 59 | Female | Absent | Lob | Right | Upper | 10.3 | Alive | - | 1.4 | 1.4 | Lepidic | INV-1 | 0 | 0 |
| 258 | 59 | Female | Absent | Lob | Right | Upper | 10.0 | Alive | - | 2.0 | 0.1 | Lepidic (MIA) | INV-2 | 0 | 0 |
| 259 | 59 | Female | Absent | Lob | Right | Upper | 6.9 | Alive | - | 1.5 | 0.4 | Lepidic (MIA) | INV-2 | 0 | 0 |
| 260 | 59 | Female | Absent | Lob | Right | Upper | 6.0 | Alive | - | 2.4 | 2.0 | Lepidic | INV-1 | 0 | 0 |
| 261 | 59 | Female | Absent | Lob | Right | Upper | 5.8 | Alive | - | 1.9 | 1.9 | Lepidic | INV-1 | 0 | 0 |
| 262 | 59 | Female | Absent | Lob | Right | Upper | 5.6 | Alive | - | 2.6 | 2.6 | Papillary | INV-1 | 0 | 0 |
| 263 | 59 | Female | Absent | Seg | Left | Upper | 7.8 | Alive | - | 1.9 | 0.0 | AIS | AIS | 0 | 0 |
| 264 | 59 | Female | Absent | Wed | Left | Upper | 5.0 | Alive | - | 0.9 | 0.4 | Lepidic (MIA) | INV-2 | 0 | 0 |
| 265 | 59 | Female | Absent | Wed | Right | Lower | 1.9 | Alive | - | 0.9 | 0.9 | Papillary | INV-1 | 0 | 0 |
| 266 | 59 | Female | Present | Lob | Right | Lower | 2.1 | Alive | - | 1.5 | 0.8 | Lepidic | INV-1 | 0 | 0 |
| 267 | 59 | Female | Present | Wed | Left | Lower | 3.8 | Alive | - | 1.3 | 0.0 | AIS | AIS | 0 | 0 |
| 268 | 59 | Female | Present | Wed | Right | Upper | 4.8 | Alive | - | 1.7 | 0.4 | Lepidic (MIA) | INV-1 | 0 | 0 |
| 269 | 59 | Male | Absent | Lob | Right | Upper | 5.7 | Alive | - | 1.7 | 0.9 | Lepidic | INV-2 | 0 | 0 |
| 270 | 59 | Male | Absent | Wed | Right | Lower | 5.2 | Alive | - | 0.8 | 0.0 | AIS | AIS | 0 | 0 |
| 271 | 59 | Male | Present | Lob | Left | Upper | 5.5 | Alive | - | 1.3 | 1.3 | Papillary | INV-1 | 0 | 0 |
| 272 | 59 | Male | Present | Lob | Left | Upper | 8.7 | Alive | - | 1.5 | 0.0 | AIS | AIS | 0 | 0 |
| 273 | 59 | Male | Present | Lob | Left | Upper | 5.1 | Alive | - | 2.1 | 2.1 | Acinar | INV-1 | 1 | 0 |
| 274 | 59 | Male | Present | Lob | Left | Upper | 4.9 | Alive | - | 2.5 | 2.5 | Acinar | INV-1 | 1 | 0 |
| 275 | 59 | Male | Present | Lob | Left | Upper | 1.5 | Alive | + | 1.4 | 1.4 | Papillary | INV-1 | 0 | 0 |
| 276 | 59 | Male | Present | Lob | Right | Lower | 4.6 | Alive | + | 2.0 | 1.2 | Papillary | INV-1 | 0 | 1 |
| 277 | 59 | Male | Present | Lob | Right | Middle | 4.1 | Alive | - | 1.2 | 0.6 | Lepidic | INV-1 | 0 | 0 |
| 278 | 59 | Male | Present | Lob | Right | Upper | 4.8 | Alive | - | 2.4 | 2.4 | Lepidic | INV-1 | 0 | 0 |
| 279 | 59 | Male | Present | Lob | Right | Upper | 7.3 | Alive | - | 2.1 | 0.7 | Lepidic | INV-1 | 0 | 0 |
| 280 | 59 | Male | Present | Lob | Right | Upper | 4.0 | Alive | - | 2.0 | 2.0 | Lepidic | INV-1 | 0 | 0 |
| 281 | 59 | Male | Present | Pneu | Left | Upper | 4.8 | Alive | - | 2.4 | 2.4 | Solid | INV-1 | 0 | 0 |
| 282 | 59 | Male | Present | Seg | Left | Upper | 5.1 | Alive | - | 1.4 | 0.1 | Lepidic (MIA) | INV-2 | 0 | 0 |
| 283 | 59 | Male | Present | Wed | Left | Upper | 10.2 | Alive | - | 1.8 | 1.0 | Papillary | INV-1 | 0 | 0 |
| 284 | 60 | Female | Absent | Lob | Left | Upper | 8.5 | Alive | - | 2.2 | 1.4 | Papillary | INV-1 | 0 | 0 |
| 285 | 60 | Female | Absent | Lob | Left | Upper | 5.1 | Alive | - | 2.8 | 2.8 | Micropapillary | INV-1 | 0 | 0 |
| 286 | 60 | Female | Absent | Lob | Left | Upper | 5.8 | Alive | - | 2.4 | 1.6 | Lepidic | INV-2 | 0 | 0 |
| 287 | 60 | Female | Absent | Lob | Left | Upper | 4.3 | Alive | - | 2.5 | 2.5 | Lepidic | INV-1 | 0 | 0 |
| 288 | 60 | Female | Absent | Lob | Right | Lower | 7.1 | Alive | - | 1.8 | 1.8 | Papillary | INV-1 | 0 | 0 |
| 289 | 60 | Female | Absent | Lob | Right | Lower | 7.7 | Alive | - | 2.2 | 0.8 | Lepidic | INV-1 | 0 | 0 |
| 290 | 60 | Female | Absent | Lob | Right | Lower | 6.0 | Alive | - | 2.0 | 0.0 | AIS | AIS | 0 | 0 |
| 291 | 60 | Female | Absent | Lob | Right | Upper | 6.3 | Alive | - | 2.5 | 2.5 | Lepidic | INV-1 | 0 | 0 |
| 292 | 60 | Female | Absent | Lob | Right | Upper | 6.5 | Alive | - | 2.4 | 0.3 | Lepidic (MIA) | INV-2 | 0 | 0 |
| 293 | 60 | Female | Absent | Lob | Right | Upper | 8.4 | Alive | - | 1.8 | 1.2 | Lepidic | INV-2 | 0 | 0 |
| 294 | 60 | Female | Absent | Lob | Right | Upper | 2.9 | Dead | + | 2.9 | 2.7 | Papillary | INV-1 | 0 | 1 |
| 295 | 60 | Female | Absent | Lob | Right | Upper | 6.8 | Alive | - | 1.9 | 0.3 | Lepidic (MIA) | INV-2 | 0 | 0 |
| 296 | 60 | Female | Absent | Seg | Left | Upper | 8.3 | Alive | - | 1.9 | 0.6 | Lepidic | INV-2 | 0 | 0 |
| 297 | 60 | Female | Absent | Seg | Left | Upper | 5.7 | Alive | - | 1.3 | 0.9 | Lepidic | INV-2 | 0 | 0 |
| 298 | 60 | Female | Absent | Seg | Left | Upper | 10.2 | Alive | - | 2.5 | 0.0 | AIS | AIS | 0 | 0 |
| 299 | 60 | Female | Absent | Seg | Left | Upper | 7.4 | Alive | - | 1.2 | 0.3 | Lepidic (MIA) | INV-1 | 0 | 0 |
| 300 | 60 | Female | Absent | Seg | Right | Lower | 5.5 | Alive | - | 0.9 | 0.0 | AIS | AIS | 0 | 0 |
| 301 | 60 | Female | Absent | Seg | Right | Lower | 10.2 | Alive | - | 1.9 | 0.0 | AIS | AIS | 0 | 0 |
| 302 | 60 | Female | Absent | Seg | Right | Lower | 5.5 | Alive | - | 1.9 | 0.5 | Lepidic (MIA) | INV-1 | 0 | 0 |
| 303 | 60 | Female | Absent | Wed | Left | Upper | 5.5 | Alive | - | 1.2 | 0.0 | AIS | AIS | 0 | 0 |
| 304 | 60 | Female | Absent | Wed | Left | Upper | 5.7 | Alive | - | 1.0 | 0.0 | AIS | AIS | 0 | 0 |
| 305 | 60 | Female | Absent | Wed | Left | Upper | 5.0 | Alive | - | 1.8 | 0.0 | AIS | AIS | 0 | 0 |
| 306 | 60 | Female | Absent | Wed | Right | Upper | 5.9 | Alive | - | 0.9 | 0.2 | Lepidic (MIA) | INV-2 | 0 | 0 |
| 307 | 60 | Female | Present | Lob | Right | Upper | 10.5 | Alive | - | 1.8 | 0.8 | Lepidic | INV-1 | 0 | 0 |
| 308 | 60 | Female | Present | Lob | Right | Upper | 5.9 | Alive | - | 2.2 | 0.2 | Lepidic (MIA) | INV-1 | 0 | 0 |
| 309 | 60 | Female | Present | Lob | Right | Upper | 5.1 | Alive | - | 1.3 | 1.3 | Solid | INV-1 | 0 | 1 |
| 310 | 60 | Male | Absent | Lob | Right | Upper | 8.1 | Alive | + | 2.2 | 1.9 | Lepidic | INV-1 | 0 | 0 |
| 311 | 60 | Male | Absent | Lob | Right | Upper | 5.1 | Alive | - | 2.3 | 0.9 | Lepidic | INV-1 | 0 | 0 |
| 312 | 60 | Male | Absent | Seg | Left | Upper | 5.3 | Alive | - | 1.7 | 0.0 | AIS | AIS | 0 | 0 |
| 313 | 60 | Male | Present | Lob | Left | Lower | 2.5 | Alive | - | 1.6 | 1.2 | Papillary | INV-1 | 0 | 0 |
| 314 | 60 | Male | Present | Lob | Left | Upper | 3.4 | Alive | - | 1.3 | 1.3 | Solid | INV-1 | 1 | 1 |
| 315 | 60 | Male | Present | Lob | Left | Upper | 5.6 | Alive | - | 2.5 | 2.5 | Papillary | INV-1 | 0 | 1 |
| 316 | 60 | Male | Present | Lob | Right | Lower | 8.1 | Alive | - | 1.5 | 1.3 | Acinar | INV-1 | 1 | 0 |
| 317 | 60 | Male | Present | Lob | Right | Lower | 5.3 | Alive | - | 1.9 | 1.0 | Lepidic | INV-1 | 0 | 0 |
| 318 | 60 | Male | Present | Lob | Right | Lower | 4.7 | Alive | - | 1.5 | 1.5 | Acinar | INV-1 | 0 | 1 |
| 319 | 60 | Male | Present | Lob | Right | Middle | 11.7 | Alive | - | 2.8 | 2.8 | Solid | INV-1 | 0 | 1 |
| 320 | 60 | Male | Present | Lob | Right | Middle | 4.3 | Alive | - | 1.2 | 0.0 | AIS | AIS | 0 | 0 |
| 321 | 60 | Male | Present | Lob | Right | Upper | 4.3 | Alive | - | 2.5 | 0.3 | Lepidic (MIA) | INV-2 | 0 | 0 |
| 322 | 60 | Male | Present | Lob | Right | Upper | 7.5 | Alive | - | 2.1 | 2.1 | Acinar | INV-1 | 0 | 1 |
| 323 | 60 | Male | Present | Lob | Right | Upper | 5.5 | Alive | - | 1.9 | 1.0 | Lepidic | INV-1 | 0 | 0 |
| 324 | 60 | Male | Present | Lob | Right | Upper | 4.2 | Alive | - | 3.0 | 3.0 | Papillary | INV-1 | 0 | 0 |
| 325 | 60 | Male | Present | Lob | Right | Upper | 5.5 | Alive | - | 2.3 | 2.3 | Papillary | INV-1 | 0 | 0 |
| 326 | 60 | Male | Present | Wed | Right | Lower | 4.3 | Alive | - | 1.5 | 1.5 | Lepidic | INV-1 | 0 | 0 |
| 327 | 61 | Female | Absent | Lob | Left | Lower | 4.3 | Alive | - | 1.8 | 0.5 | Lepidic (MIA) | INV-1 | 0 | 0 |
| 328 | 61 | Female | Absent | Lob | Left | Lower | 8.8 | Alive | - | 1.8 | 1.0 | Papillary | INV-1 | 0 | 0 |
| 329 | 61 | Female | Absent | Lob | Left | Upper | 5.0 | Alive | - | 2.7 | 2.7 | Variant | INV-1 | 0 | 0 |
| 330 | 61 | Female | Absent | Lob | Right | Middle | 9.3 | Alive | - | 1.4 | 0.0 | AIS | AIS | 0 | 0 |
| 331 | 61 | Female | Absent | Lob | Right | Upper | 4.0 | Alive | - | 1.8 | 1.0 | Lepidic | INV-2 | 0 | 0 |
| 332 | 61 | Female | Absent | Lob | Right | Upper | 5.1 | Alive | - | 1.5 | 0.6 | Lepidic | INV-2 | 0 | 0 |
| 333 | 61 | Female | Absent | Seg | Left | Lower | 8.0 | Alive | - | 1.6 | 0.0 | AIS | AIS | 0 | 0 |
| 334 | 61 | Female | Absent | Seg | Left | Lower | 5.3 | Alive | - | 1.4 | 1.1 | Lepidic | INV-1 | 0 | 0 |
| 335 | 61 | Female | Absent | Seg | Right | Lower | 5.0 | Alive | - | 1.9 | 0.5 | Lepidic (MIA) | INV-2 | 0 | 0 |
| 336 | 61 | Female | Absent | Wed | Right | Lower | 5.5 | Alive | - | 1.3 | 0.0 | AIS | AIS | 0 | 0 |
| 337 | 61 | Female | Absent | Wed | Right | Lower | 4.5 | Alive | - | 1.5 | 0.0 | AIS | AIS | 0 | 0 |
| 338 | 61 | Female | Absent | Wed | Right | Upper | 5.5 | Alive | - | 1.5 | 0.0 | AIS | AIS | 0 | 0 |
| 339 | 61 | Female | Present | Lob | Right | Lower | 8.5 | Alive | - | 1.8 | 1.2 | Papillary | INV-1 | 1 | 1 |
| 340 | 61 | Female | Present | Lob | Right | Lower | 4.9 | Alive | - | 2.5 | 1.8 | Lepidic | INV-1 | 0 | 0 |
| 341 | 61 | Female | Present | Seg | Left | Lower | 6.1 | Alive | - | 0.9 | 0.1 | Lepidic (MIA) | INV-1 | 0 | 0 |
| 342 | 61 | Female | Present | Wed | Right | Upper | 0.6 | Dead | + | 1.4 | 1.4 | Micropapillary | INV-1 | 0 | 1 |
| 343 | 61 | Female | Present | Wed | Right | Upper | 4.5 | Alive | - | 0.8 | 0.0 | AIS | AIS | 0 | 0 |
| 344 | 61 | Female |  | Lob | Right | Lower | 5.0 | Alive | - | 1.2 | 0.6 | Lepidic | INV-1 | 0 | 0 |
| 345 | 61 | Female |  | Wed | Right | Lower | 6.8 | Alive | - | 2.2 | 0.2 | Lepidic (MIA) | INV-1 | 0 | 0 |
| 346 | 61 | Male | Absent | Lob | Left | Upper | 6.0 | Alive | - | 1.4 | 1.4 | Lepidic | INV-1 | 0 | 0 |
| 347 | 61 | Male | Absent | Lob | Right | Upper | 6.9 | Alive | - | 1.2 | 0.2 | Lepidic (MIA) | INV-1 | 0 | 0 |
| 348 | 61 | Male | Absent | Seg | Left | Upper | 3.8 | Alive | - | 2.2 | 0.4 | Lepidic (MIA) | INV-1 | 0 | 0 |
| 349 | 61 | Male | Absent | Seg | Right | Lower | 2.6 | Alive | - | 2.4 | 2.4 | Papillary | INV-1 | 0 | 0 |
| 350 | 61 | Male | Absent | Seg | Right | Lower | 9.5 | Alive | - | 1.0 | 1.0 | Papillary | INV-1 | 0 | 0 |
| 351 | 61 | Male | Absent | Wed | Left | Lower | 6.6 | Alive | - | 1.1 | 1.1 | Lepidic | INV-2 | 0 | 0 |
| 352 | 61 | Male | Present | Lob | Left | Lower | 4.0 | Alive | - | 3.0 | 3.0 | Papillary | INV-1 | 0 | 0 |
| 353 | 61 | Male | Present | Lob | Left | Upper | 5.1 | Alive | - | 2.2 | 1.0 | Lepidic | INV-1 | 0 | 0 |
| 354 | 61 | Male | Present | Lob | Left | Upper | 5.6 | Alive | - | 1.3 | 1.3 | Papillary | INV-1 | 0 | 0 |
| 355 | 61 | Male | Present | Lob | Right | Lower | 8.8 | Alive | - | 1.8 | 0.3 | Lepidic (MIA) | INV-1 | 0 | 0 |
| 356 | 61 | Male | Present | Lob | Right | Lower | 5.0 | Alive | - | 1.0 | 0.8 | Papillary | INV-1 | 0 | 0 |
| 357 | 61 | Male | Present | Lob | Right | Middle | 4.9 | Alive | - | 1.9 | 0.0 | AIS | AIS | 0 | 0 |
| 358 | 61 | Male | Present | Lob | Right | Upper | 8.3 | Alive | - | 1.4 | 1.4 | Lepidic | INV-1 | 0 | 1 |
| 359 | 61 | Male | Present | Lob | Right | Upper | 6.0 | Alive | - | 1.0 | 0.6 | Lepidic | INV-2 | 0 | 0 |
| 360 | 61 | Male | Present | Lob | Right | Upper | 2.0 | Alive | - | 2.2 | 2.2 | Acinar | INV-1 | 0 | 0 |
| 361 | 61 | Male | Present | Seg | Left | Upper | 9.1 | Alive | - | 2.0 | 0.3 | Lepidic (MIA) | INV-2 | 0 | 0 |
| 362 | 61 | Male | Present | Seg | Right | Lower | 5.4 | Alive | - | 1.8 | 1.8 | Lepidic | INV-1 | 0 | 0 |
| 363 | 61 | Male | Present | Seg | Right | Lower | 5.5 | Alive | - | 1.4 | 1.0 | Lepidic | INV-2 | 0 | 0 |
| 364 | 61 | Male | Present | Wed | Left | Lower | 5.4 | Alive | - | 2.1 | 0.2 | Lepidic (MIA) | INV-1 | 0 | 0 |
| 365 | 61 | Male | Present | Wed | Left | Upper | 4.6 | Alive | - | 1.5 | 0.0 | AIS | AIS | 0 | 0 |
| 366 | 62 | Female | Absent | Lob | Left | Lower | 3.1 | Dead | + | 2.2 | 2.2 | Solid | INV-1 | 1 | 1 |
| 367 | 62 | Female | Absent | Lob | Left | Upper | 7.9 | Alive | - | 1.6 | 0.9 | Lepidic | INV-2 | 0 | 0 |
| 368 | 62 | Female | Absent | Lob | Right | Lower | 4.6 | Alive | - | 0.9 | 0.9 | Acinar | INV-1 | 1 | 0 |
| 369 | 62 | Female | Absent | Lob | Right | Lower | 8.4 | Alive | - | 2.2 | 2.2 | Lepidic | INV-1 | 0 | 0 |
| 370 | 62 | Female | Absent | Lob | Right | Middle | 11.1 | Alive | - | 1.4 | 0.2 | Lepidic (MIA) | INV-2 | 0 | 0 |
| 371 | 62 | Female | Absent | Lob | Right | Middle | 6.1 | Alive | - | 1.7 | 0.0 | AIS | AIS | 0 | 0 |
| 372 | 62 | Female | Absent | Lob | Right | Upper | 5.7 | Alive | - | 2.2 | 2.2 | Papillary | INV-1 | 0 | 0 |
| 373 | 62 | Female | Absent | Lob | Right | Upper | 4.6 | Alive | - | 2.3 | 0.8 | Lepidic | INV-2 | 0 | 0 |
| 374 | 62 | Female | Absent | Seg | Left | Lower | 7.7 | Alive | - | 2.7 | 0.5 | Lepidic (MIA) | INV-2 | 0 | 0 |
| 375 | 62 | Female | Absent | Seg | Left | Upper | 4.1 | Alive | - | 2.0 | 0.0 | AIS | AIS | 0 | 0 |
| 376 | 62 | Female | Absent | Seg | Right | Upper | 4.9 | Alive | - | 1.5 | 1.5 | Papillary | INV-1 | 0 | 0 |
| 377 | 62 | Female | Absent | Wed | Left | Upper | 6.3 | Alive | - | 1.1 | 0.2 | Lepidic (MIA) | INV-1 | 0 | 0 |
| 378 | 62 | Female | Absent | Wed | Right | Lower | 4.0 | Alive | - | 1.3 | 0.0 | AIS | AIS | 0 | 0 |
| 379 | 62 | Female | Absent | Wed | Right | Upper | 6.0 | Alive | - | 1.2 | 0.0 | AIS | AIS | 0 | 0 |
| 380 | 62 | Female | Absent | Wed | Right | Upper | 6.1 | Alive | - | 1.0 | 0.3 | Lepidic (MIA) | INV-1 | 0 | 0 |
| 381 | 62 | Female | Present | Lob | Left | Lower | 8.0 | Alive | - | 2.9 | 2.1 | Lepidic | INV-1 | 0 | 0 |
| 382 | 62 | Female | Present | Lob | Left | Lower | 5.5 | Alive | - | 2.3 | 2.3 | Papillary | INV-1 | 0 | 0 |
| 383 | 62 | Female | Present | Lob | Left | Upper | 10.6 | Alive | - | 2.2 | 2.2 | Papillary | INV-1 | 0 | 0 |
| 384 | 62 | Female | Present | Lob | Left | Upper | 3.0 | Alive | + | 2.4 | 2.4 | Papillary | INV-1 | 0 | 0 |
| 385 | 62 | Female | Present | Lob | Left | Upper | 7.1 | Alive | - | 1.5 | 0.0 | AIS | AIS | 0 | 0 |
| 386 | 62 | Female | Present | Lob | Left | Upper | 4.5 | Alive | - | 1.3 | 1.3 | Papillary | INV-1 | 0 | 0 |
| 387 | 62 | Female | Present | Lob | Right | Upper | 7.4 | Alive | - | 1.8 | 1.4 | Lepidic | INV-1 | 0 | 0 |
| 388 | 62 | Female | Present | Lob | Right | Upper | 4.7 | Alive | - | 1.2 | 0.7 | Lepidic | INV-2 | 0 | 0 |
| 389 | 62 | Female | Present | Wed | Left | Upper | 6.0 | Alive | - | 0.9 | 0.8 | Lepidic | INV-2 | 0 | 0 |
| 390 | 62 | Male | Absent | Lob | Left | Lower | 8.9 | Alive | - | 1.8 | 1.8 | Acinar | INV-1 | 0 | 0 |
| 391 | 62 | Male | Absent | Lob | Left | Lower | 6.1 | Alive | - | 2.2 | 2.2 | Papillary | INV-1 | 0 | 0 |
| 392 | 62 | Male | Present | Lob | Right | Lower | 3.8 | Alive | - | 1.0 | 1.0 | Lepidic | INV-1 | 0 | 0 |
| 393 | 62 | Male | Present | Lob | Right | Middle | 7.8 | Alive | - | 1.5 | 0.8 | Lepidic | INV-1 | 0 | 0 |
| 394 | 62 | Male | Present | Lob | Right | Upper | 9.4 | Alive | - | 2.1 | 1.2 | Lepidic | INV-2 | 0 | 0 |
| 395 | 62 | Male | Present | Lob | Right | Upper | 5.0 | Alive | - | 1.2 | 0.7 | Lepidic | INV-2 | 0 | 0 |
| 396 | 62 | Male | Present | Lob | Right | Upper | 2.1 | Dead | - | 2.6 | 0.0 | AIS | AIS | 0 | 0 |
| 397 | 62 | Male | Present | Lob | Right | Upper | 2.1 | Dead | - | 1.5 | 1.5 | Lepidic | INV-2 | 0 | 0 |
| 398 | 62 | Male | Present | Lob | Right | Upper | 5.0 | Alive | - | 1.7 | 1.7 | Variant | INV-1 | 0 | 0 |
| 399 | 62 | Male | Present | Seg | Left | Upper | 9.8 | Alive | - | 1.9 | 0.3 | Lepidic (MIA) | INV-1 | 0 | 0 |
| 400 | 62 | Male | Present | Wed | Left | Upper | 8.4 | Alive | + | 1.5 | 1.5 | Solid | INV-1 | 1 | 1 |
| 401 | 62 | Male | Present | Wed | Right | Upper | 7.5 | Alive | - | 1.0 | 1.0 | Solid | INV-1 | 0 | 1 |
| 402 | 62 | Male |  | Seg | Right | Lower | 5.2 | Alive | - | 2.0 | 2.0 | Lepidic | INV-1 | 0 | 0 |
| 403 | 63 | Female | Absent | Lob | Left | Lower | 5.2 | Alive | - | 1.6 | 1.6 | Acinar | INV-1 | 0 | 0 |
| 404 | 63 | Female | Absent | Lob | Left | Lower | 3.9 | Alive | - | 1.5 | 0.6 | Lepidic | INV-1 | 0 | 0 |
| 405 | 63 | Female | Absent | Lob | Left | Upper | 3.3 | Alive | - | 2.2 | 2.2 | Lepidic | INV-1 | 1 | 0 |
| 406 | 63 | Female | Absent | Lob | Left | Upper | 8.5 | Alive | - | 1.9 | 1.9 | Lepidic | INV-1 | 0 | 0 |
| 407 | 63 | Female | Absent | Lob | Left | Upper | 4.9 | Alive | - | 1.3 | 0.1 | Lepidic (MIA) | INV-1 | 0 | 0 |
| 408 | 63 | Female | Absent | Lob | Left | Upper | 5.0 | Alive | - | 2.0 | 0.2 | Lepidic (MIA) | INV-2 | 0 | 0 |
| 409 | 63 | Female | Absent | Lob | Right | Lower | 4.5 | Alive | - | 2.9 | 1.1 | Lepidic | INV-1 | 0 | 0 |
| 410 | 63 | Female | Absent | Lob | Right | Lower | 4.4 | Alive | - | 1.9 | 1.9 | Papillary | INV-1 | 0 | 0 |
| 411 | 63 | Female | Absent | Lob | Right | Upper | 5.5 | Alive | - | 2.6 | 0.4 | Lepidic (MIA) | INV-2 | 0 | 0 |
| 412 | 63 | Female | Absent | Lob | Right | Upper | 8.3 | Alive | - | 2.1 | 0.2 | Lepidic (MIA) | INV-1 | 0 | 0 |
| 413 | 63 | Female | Absent | Lob | Right | Upper | 5.1 | Alive | - | 1.3 | 0.9 | Lepidic | INV-1 | 0 | 0 |
| 414 | 63 | Female | Absent | Lob | Right | Upper | 5.6 | Alive | - | 2.9 | 0.3 | Lepidic (MIA) | INV-2 | 0 | 0 |
| 415 | 63 | Female | Absent | Seg | Left | Upper | 9.3 | Alive | - | 1.5 | 0.8 | Lepidic | INV-2 | 0 | 0 |
| 416 | 63 | Female | Absent | Seg | Left | Upper | 5.6 | Alive | - | 1.6 | 0.7 | Lepidic | INV-2 | 0 | 0 |
| 417 | 63 | Female | Absent | Seg | Right | Lower | 5.1 | Alive | - | 1.5 | 0.4 | Lepidic (MIA) | INV-1 | 0 | 0 |
| 418 | 63 | Female | Absent | Seg | Right | Upper | 4.5 | Alive | - | 0.5 | 0.0 | AIS | AIS | 0 | 0 |
| 419 | 63 | Female | Absent | Seg | Right | Upper | 9.3 | Alive | - | 1.8 | 0.3 | Lepidic (MIA) | INV-1 | 0 | 0 |
| 420 | 63 | Female | Absent | Wed | Right | Upper | 4.7 | Alive | - | 1.3 | 0.0 | AIS | AIS | 0 | 0 |
| 421 | 63 | Female | Absent | Wed | Right | Upper | 5.5 | Alive | - | 1.0 | 0.0 | AIS | AIS | 0 | 0 |
| 422 | 63 | Female | Present | Lob | Right | Upper | 4.0 | Alive | - | 1.2 | 1.2 | Solid | INV-1 | 0 | 1 |
| 423 | 63 | Female | Present | Lob | Right | Upper | 5.6 | Alive | - | 2.0 | 0.8 | Lepidic | INV-1 | 0 | 0 |
| 424 | 63 | Female |  | Lob | Right | Middle | 5.1 | Alive | - | 1.8 | 1.8 | Acinar | INV-1 | 0 | 0 |
| 425 | 63 | Male | Absent | Lob | Right | Middle | 4.2 | Alive | - | 2.3 | 1.7 | Papillary | INV-1 | 0 | 0 |
| 426 | 63 | Male | Absent | Wed | Left | Lower | 6.0 | Alive | - | 1.8 | 0.1 | Lepidic (MIA) | INV-1 | 0 | 0 |
| 427 | 63 | Male | Present | Lob | Left | Lower | 5.3 | Alive | - | 1.9 | 1.9 | Variant | INV-1 | 0 | 0 |
| 428 | 63 | Male | Present | Lob | Left | Upper | 3.5 | Alive | + | 1.2 | 1.2 | Papillary | INV-1 | 0 | 0 |
| 429 | 63 | Male | Present | Lob | Right | Lower | 9.4 | Alive | - | 2.1 | 2.1 | Solid | INV-1 | 0 | 1 |
| 430 | 63 | Male | Present | Lob | Right | Upper | 6.5 | Alive | - | 1.4 | 0.9 | Lepidic | INV-2 | 0 | 0 |
| 431 | 63 | Male | Present | Lob | Right | Upper | 5.0 | Alive | - | 2.3 | 2.3 | Papillary | INV-1 | 0 | 0 |
| 432 | 63 | Male | Present | Lob | Right | Upper | 4.9 | Dead | - | 3.0 | 2.8 | Papillary | INV-1 | 1 | 1 |
| 433 | 63 | Male | Present | Lob | Right | Upper | 3.0 | Dead | - | 1.9 | 1.9 | Papillary | INV-1 | 0 | 0 |
| 434 | 63 | Male | Present | Wed | Right | Upper | 5.8 | Alive | - | 1.1 | 0.6 | Acinar | INV-1 | 0 | 0 |
| 435 | 63 | Male | Present | Wed | Right | Upper | 4.5 | Alive | - | 1.8 | 0.0 | AIS | AIS | 0 | 0 |
| 436 | 63 | Male |  | Seg | Left | Upper | 5.2 | Alive | - | 1.5 | 1.1 | Lepidic | INV-1 | 0 | 0 |
| 437 | 64 | Female | Absent | Lob | Left | Lower | 6.3 | Alive | - | 1.8 | 0.8 | Lepidic | INV-1 | 0 | 0 |
| 438 | 64 | Female | Absent | Lob | Left | Upper | 7.1 | Alive | - | 2.5 | 2.2 | Solid | INV-1 | 0 | 1 |
| 439 | 64 | Female | Absent | Lob | Right | Lower | 10.0 | Alive | - | 1.2 | 1.2 | Lepidic | INV-1 | 1 | 0 |
| 440 | 64 | Female | Absent | Lob | Right | Lower | 8.9 | Alive | - | 1.5 | 1.0 | Lepidic | INV-1 | 0 | 0 |
| 441 | 64 | Female | Absent | Lob | Right | Middle | 6.8 | Alive | - | 2.7 | 2.7 | Acinar | INV-1 | 0 | 0 |
| 442 | 64 | Female | Absent | Lob | Right | Upper | 3.2 | Alive | - | 1.8 | 0.6 | Lepidic | INV-1 | 0 | 0 |
| 443 | 64 | Female | Absent | Lob | Right | Upper | 6.4 | Alive | - | 2.5 | 1.2 | Lepidic | INV-2 | 0 | 0 |
| 444 | 64 | Female | Absent | Lob | Right | Upper | 4.8 | Alive | - | 1.9 | 1.9 | Lepidic | INV-1 | 0 | 0 |
| 445 | 64 | Female | Absent | Lob | Right | Upper | 4.0 | Alive | - | 1.5 | 1.5 | Acinar | INV-1 | 0 | 0 |
| 446 | 64 | Female | Absent | Seg | Left | Upper | 7.3 | Alive | - | 2.1 | 0.6 | Lepidic | INV-1 | 0 | 0 |
| 447 | 64 | Female | Absent | Seg | Right | Lower | 3.8 | Alive | - | 1.7 | 1.6 | Lepidic | INV-1 | 0 | 0 |
| 448 | 64 | Female | Absent | Seg | Right | Upper | 8.3 | Alive | - | 1.8 | 0.0 | AIS | AIS | 0 | 0 |
| 449 | 64 | Female | Absent | Wed | Right | Lower | 6.4 | Alive | - | 1.4 | 0.2 | Lepidic (MIA) | INV-2 | 0 | 0 |
| 450 | 64 | Female | Absent | Wed | Right | Upper | 5.4 | Alive | - | 1.5 | 0.3 | Lepidic (MIA) | INV-1 | 0 | 0 |
| 451 | 64 | Female | Present | Lob | Left | Lower | 5.6 | Alive | - | 1.8 | 1.8 | Lepidic | INV-1 | 0 | 0 |
| 452 | 64 | Female | Present | Lob | Left | Upper | 4.9 | Alive | + | 1.8 | 1.8 | Solid | INV-1 | 1 | 1 |
| 453 | 64 | Female | Present | Seg | Right | Lower | 8.3 | Alive | - | 1.4 | 0.0 | AIS | AIS | 0 | 0 |
| 454 | 64 | Female | Present | Seg | Right | Lower | 7.1 | Alive | - | 1.2 | 0.0 | AIS | AIS | 0 | 0 |
| 455 | 64 | Female | Present | Wed | Right | Lower | 7.4 | Alive | - | 1.8 | 0.0 | AIS | AIS | 0 | 0 |
| 456 | 64 | Male | Absent | Lob | Right | Upper | 7.6 | Alive | - | 1.8 | 0.9 | Lepidic | INV-2 | 0 | 0 |
| 457 | 64 | Male | Absent | Seg | Left | Lower | 5.0 | Alive | - | 1.5 | 1.5 | Lepidic | INV-1 | 0 | 0 |
| 458 | 64 | Male | Absent | Seg | Left | Upper | 6.4 | Alive | - | 2.1 | 0.5 | Lepidic (MIA) | INV-1 | 0 | 0 |
| 459 | 64 | Male | Absent | Seg | Left | Upper | 3.7 | Alive | - | 1.9 | 0.8 | Lepidic | INV-1 | 0 | 0 |
| 460 | 64 | Male | Absent | Seg | Right | Lower | 5.2 | Alive | - | 1.4 | 0.0 | AIS | AIS | 0 | 0 |
| 461 | 64 | Male | Present | Lob | Left | Upper | 3.2 | Alive | + | 2.5 | 0.5 | Lepidic (MIA) | INV-1 | 1 | 1 |
| 462 | 64 | Male | Present | Lob | Left | Upper | 10.1 | Alive | - | 1.8 | 0.8 | Papillary | INV-1 | 1 | 0 |
| 463 | 64 | Male | Present | Lob | Left | Upper | 8.4 | Alive | - | 2.1 | 2.1 | Papillary | INV-1 | 0 | 0 |
| 464 | 64 | Male | Present | Lob | Right | Lower | 8.2 | Alive | - | 2.6 | 1.0 | Lepidic | INV-2 | 0 | 0 |
| 465 | 64 | Male | Present | Lob | Right | Lower | 4.0 | Alive | - | 2.5 | 2.5 | Solid | INV-1 | 0 | 0 |
| 466 | 64 | Male | Present | Lob | Right | Middle | 7.9 | Alive | - | 1.2 | 0.7 | Papillary | INV-1 | 0 | 0 |
| 467 | 64 | Male | Present | Lob | Right | Middle | 5.3 | Alive | - | 2.5 | 2.5 | Acinar | INV-1 | 0 | 0 |
| 468 | 64 | Male | Present | Lob | Right | Upper | 7.9 | Alive | - | 2.7 | 2.7 | Papillary | INV-1 | 1 | 1 |
| 469 | 64 | Male | Present | Lob | Right | Upper | 10.8 | Alive | - | 0.9 | 0.9 | Papillary | INV-1 | 0 | 0 |
| 470 | 64 | Male | Present | Lob | Right | Upper | 7.4 | Alive | - | 1.1 | 0.0 | AIS | AIS | 0 | 0 |
| 471 | 64 | Male | Present | Lob | Right | Upper | 5.8 | Alive | - | 1.1 | 0.2 | Lepidic (MIA) | INV-1 | 0 | 0 |
| 472 | 64 | Male | Present | Lob | Right | Upper | 5.6 | Alive | - | 1.5 | 1.5 | Lepidic | INV-1 | 0 | 1 |
| 473 | 64 | Male | Present | Seg | Left | Upper | 8.4 | Alive | - | 2.8 | 2.8 | Lepidic | INV-1 | 0 | 0 |
| 474 | 64 | Male | Present | Seg | Left | Upper | 5.4 | Alive | - | 1.6 | 0.2 | Lepidic (MIA) | INV-1 | 0 | 0 |
| 475 | 64 | Male | Present | Seg | Right | Lower | 5.1 | Alive | - | 1.2 | 0.2 | Lepidic (MIA) | INV-1 | 0 | 0 |
| 476 | 64 | Male | Present | Seg | Right | Lower | 4.8 | Alive | - | 1.7 | 0.7 | Lepidic | INV-2 | 0 | 0 |
| 477 | 64 | Male | Present | Seg | Right | Upper | 4.7 | Alive | - | 1.5 | 0.1 | Lepidic (MIA) | INV-2 | 0 | 0 |
| 478 | 64 | Male | Present | Wed | Left | Lower | 6.2 | Alive | - | 1.8 | 1.8 | Papillary | INV-1 | 0 | 0 |
| 479 | 64 | Male | Present | Wed | Left | Upper | 9.3 | Alive | - | 2.2 | 1.0 | Lepidic | INV-2 | 0 | 0 |
| 480 | 64 | Male | Present | Wed | Left | Upper | 4.2 | Alive | - | 0.9 | 0.9 | Papillary | INV-1 | 0 | 1 |
| 481 | 64 | Male | Present | Wed | Right | Lower | 6.0 | Alive | - | 0.4 | 0.0 | AIS | AIS | 0 | 0 |
| 482 | 64 | Male | Present | Wed | Right | Upper | 4.0 | Alive | - | 2.5 | 1.7 | Lepidic | INV-1 | 0 | 0 |
| 483 | 65 | Female | Absent | Lob | Left | Upper | 5.3 | Alive | - | 1.5 | 1.5 | Solid | INV-1 | 1 | 1 |
| 484 | 65 | Female | Absent | Lob | Right | Lower | 5.7 | Alive | - | 1.4 | 0.4 | Lepidic (MIA) | INV-1 | 0 | 0 |
| 485 | 65 | Female | Absent | Lob | Right | Lower | 5.5 | Alive | - | 1.2 | 0.3 | Lepidic (MIA) | INV-1 | 0 | 0 |
| 486 | 65 | Female | Absent | Lob | Right | Lower | 6.1 | Alive | - | 2.5 | 2.5 | Variant | INV-1 | 0 | 0 |
| 487 | 65 | Female | Absent | Lob | Right | Middle | 7.4 | Alive | - | 2.6 | 1.0 | Lepidic | INV-2 | 0 | 0 |
| 488 | 65 | Female | Absent | Lob | Right | Upper | 3.7 | Alive | - | 1.5 | 0.1 | Lepidic (MIA) | INV-2 | 0 | 0 |
| 489 | 65 | Female | Absent | Seg | Left | Upper | 8.0 | Alive | - | 1.3 | 1.3 | Papillary | INV-1 | 0 | 1 |
| 490 | 65 | Female | Absent | Seg | Right | Lower | 5.5 | Alive | - | 1.9 | 1.9 | Lepidic | INV-1 | 0 | 0 |
| 491 | 65 | Female | Absent | Seg | Right | Lower | 6.6 | Alive | - | 2.9 | 0.0 | AIS | AIS | 0 | 0 |
| 492 | 65 | Female | Absent | Wed | Left | Lower | 5.3 | Alive | - | 1.5 | 1.5 | Papillary | INV-1 | 0 | 0 |
| 493 | 65 | Female | Absent | Wed | Left | Upper | 6.4 | Alive | - | 0.5 | 0.0 | AIS | AIS | 0 | 0 |
| 494 | 65 | Female | Absent | Wed | Left | Upper | 5.5 | Alive | - | 2.2 | 0.7 | Lepidic | INV-2 | 0 | 0 |
| 495 | 65 | Female | Absent | Wed | Right | Lower | 5.6 | Alive | - | 0.7 | 0.0 | AIS | AIS | 0 | 0 |
| 496 | 65 | Female | Absent | Wed | Right | Upper | 5.7 | Alive | - | 1.3 | 0.7 | Lepidic | INV-2 | 0 | 0 |
| 497 | 65 | Female | Present | Lob | Right | Upper | 5.1 | Alive | - | 1.9 | 0.0 | AIS | AIS | 0 | 0 |
| 498 | 65 | Female |  | Lob | Right | Upper | 4.7 | Alive | - | 2.8 | 0.6 | Lepidic | INV-1 | 0 | 0 |
| 499 | 65 | Male | Present | Lob | Left | Lower | 5.1 | Alive | - | 1.2 | 1.2 | Micropapillary | INV-1 | 0 | 0 |
| 500 | 65 | Male | Present | Lob | Left | Lower | 4.8 | Alive | - | 0.9 | 0.9 | Papillary | INV-1 | 0 | 0 |
| 501 | 65 | Male | Present | Lob | Right | Lower | 10.1 | Alive | - | 2.5 | 2.5 | Papillary | INV-1 | 1 | 1 |
| 502 | 65 | Male | Present | Lob | Right | Lower | 4.3 | Alive | - | 2.7 | 2.7 | Papillary | INV-1 | 1 | 1 |
| 503 | 65 | Male | Present | Lob | Right | Upper | 9.0 | Alive | - | 2.0 | 0.1 | Lepidic (MIA) | INV-2 | 0 | 0 |
| 504 | 65 | Male | Present | Lob | Right | Upper | 6.1 | Alive | - | 2.1 | 2.1 | Lepidic | INV-1 | 0 | 1 |
| 505 | 65 | Male | Present | Lob | Right | Upper | 6.2 | Alive | - | 1.5 | 0.0 | AIS | AIS | 0 | 0 |
| 506 | 65 | Male | Present | Lob | Right | Upper | 6.2 | Alive | - | 2.2 | 0.7 | Lepidic | INV-2 | 0 | 0 |
| 507 | 65 | Male | Present | Lob | Right | Upper | 4.6 | Alive | - | 2.7 | 2.7 | Acinar | INV-1 | 0 | 1 |
| 508 | 65 | Male | Present | Lob | Right | Upper | 4.0 | Alive | - | 2.5 | 0.2 | Lepidic (MIA) | INV-2 | 0 | 0 |
| 509 | 65 | Male | Present | Lob | Right | Upper | 5.9 | Alive | - | 2.4 | 0.3 | Lepidic (MIA) | INV-2 | 0 | 0 |
| 510 | 65 | Male | Present | Lob | Right | Upper | 5.1 | Alive | - | 1.8 | 1.0 | Lepidic | INV-2 | 0 | 0 |
| 511 | 65 | Male | Present | Seg | Left | Lower | 1.8 | Dead | - | 1.8 | 0.5 | Lepidic (MIA) | INV-1 | 1 | 0 |
| 512 | 65 | Male | Present | Seg | Right | Upper | 7.1 | Alive | - | 0.8 | 0.8 | Papillary | INV-1 | 0 | 0 |
| 513 | 65 | Male | Present | Seg | Right | Upper | 9.4 | Alive | - | 1.4 | 0.6 | Lepidic | INV-1 | 0 | 0 |
| 514 | 65 | Male | Present | Wed | Left | Lower | 5.5 | Alive | - | 1.2 | 1.2 | Papillary | INV-1 | 0 | 0 |
| 515 | 65 | Male | Present | Wed | Left | Lower | 5.5 | Alive | - | 1.5 | 0.0 | AIS | AIS | 0 | 0 |
| 516 | 65 | Male | Present | Wed | Left | Lower | 6.0 | Alive | - | 2.3 | 2.3 | Papillary | INV-1 | 0 | 0 |
| 517 | 65 | Male | Present | Wed | Left | Upper | 8.5 | Alive | - | 1.2 | 0.3 | Lepidic (MIA) | INV-2 | 0 | 0 |
| 518 | 65 | Male | Present | Wed | Right | Lower | 5.9 | Alive | - | 1.4 | 1.0 | Lepidic | INV-1 | 0 | 0 |
| 519 | 65 | Male |  | Lob | Right | Upper | 5.8 | Alive | - | 2.5 | 1.0 | Lepidic | INV-2 | 0 | 0 |
| 520 | 65 | Male |  | Lob | Right | Upper | 8.5 | Alive | - | 2.5 | 2.5 | Papillary | INV-1 | 0 | 0 |
| 521 | 66 | Female | Absent | Lob | Left | Lower | 6.1 | Alive | - | 1.7 | 1.7 | Papillary | INV-1 | 1 | 1 |
| 522 | 66 | Female | Absent | Lob | Left | Upper | 8.5 | Alive | - | 2.7 | 2.5 | Papillary | INV-1 | 1 | 0 |
| 523 | 66 | Female | Absent | Lob | Right | Middle | 6.2 | Alive | - | 1.0 | 0.0 | AIS | AIS | 0 | 0 |
| 524 | 66 | Female | Absent | Lob | Right | Upper | 6.0 | Alive | - | 2.8 | 2.8 | Papillary | INV-1 | 1 | 1 |
| 525 | 66 | Female | Absent | Lob | Right | Upper | 8.5 | Alive | - | 2.8 | 2.8 | Papillary | INV-1 | 1 | 0 |
| 526 | 66 | Female | Absent | Lob | Right | Upper | 8.3 | Alive | - | 2.8 | 0.8 | Lepidic | INV-2 | 0 | 0 |
| 527 | 66 | Female | Absent | Lob | Right | Upper | 5.1 | Alive | - | 0.8 | 0.0 | AIS | AIS | 0 | 0 |
| 528 | 66 | Female | Absent | Lob | Right | Upper | 4.6 | Alive | - | 2.4 | 0.3 | Lepidic (MIA) | INV-1 | 0 | 0 |
| 529 | 66 | Female | Absent | Lob | Right | Upper | 4.1 | Alive | - | 1.8 | 1.8 | Acinar | INV-1 | 0 | 0 |
| 530 | 66 | Female | Absent | Lob | Right | Upper | 6.0 | Alive | - | 1.5 | 1.5 | Lepidic | INV-1 | 0 | 0 |
| 531 | 66 | Female | Absent | Lob | Right | Upper | 4.8 | Alive | - | 2.1 | 0.5 | Lepidic (MIA) | INV-2 | 0 | 0 |
| 532 | 66 | Female | Absent | Seg | Left | Upper | 2.7 | Dead | - | 2.7 | 0.5 | Lepidic (MIA) | INV-1 | 0 | 0 |
| 533 | 66 | Female | Absent | Seg | Left | Upper | 6.8 | Alive | - | 1.8 | 0.2 | Lepidic (MIA) | INV-2 | 0 | 0 |
| 534 | 66 | Female | Absent | Seg | Left | Upper | 6.1 | Alive | - | 1.7 | 0.3 | Lepidic (MIA) | INV-2 | 0 | 0 |
| 535 | 66 | Female | Absent | Seg | Right | Lower | 5.0 | Alive | - | 1.8 | 0.8 | Lepidic | INV-2 | 0 | 0 |
| 536 | 66 | Female | Absent | Seg | Right | Lower | 4.0 | Alive | + | 1.5 | 0.3 | Lepidic (MIA) | INV-2 | 0 | 0 |
| 537 | 66 | Female | Absent | Wed | Left | Lower | 6.1 | Alive | - | 1.8 | 1.8 | Papillary | INV-1 | 0 | 1 |
| 538 | 66 | Female | Absent | Wed | Left | Lower | 4.0 | Alive | - | 0.8 | 0.3 | Lepidic (MIA) | INV-1 | 0 | 0 |
| 539 | 66 | Female | Absent | Wed | Right | Upper | 5.0 | Alive | - | 0.7 | 0.0 | AIS | AIS | 0 | 0 |
| 540 | 66 | Female | Absent | Wed | Right | Upper | 4.9 | Alive | - | 1.4 | 0.0 | AIS | AIS | 0 | 0 |
| 541 | 66 | Female | Absent | Wed | Right | Upper | 4.8 | Alive | - | 1.8 | 0.0 | AIS | AIS | 0 | 0 |
| 542 | 66 | Female | Present | Lob | Right | Upper | 5.8 | Alive | - | 2.1 | 2.1 | Papillary | INV-1 | 0 | 0 |
| 543 | 66 | Female | Present | Seg | Left | Lower | 5.2 | Alive | - | 2.3 | 0.7 | Lepidic | INV-2 | 0 | 0 |
| 544 | 66 | Male | Present | Lob | Left | Lower | 5.2 | Alive | - | 2.2 | 1.4 | Lepidic | INV-1 | 0 | 0 |
| 545 | 66 | Male | Present | Lob | Right | Middle | 2.7 | Alive | - | 0.9 | 0.0 | AIS | AIS | 0 | 0 |
| 546 | 66 | Male | Present | Lob | Right | Upper | 10.4 | Alive | - | 1.4 | 1.4 | Solid | INV-1 | 0 | 1 |
| 547 | 66 | Male | Present | Lob | Right | Upper | 4.8 | Alive | - | 1.6 | 1.6 | Lepidic | INV-1 | 0 | 0 |
| 548 | 66 | Male | Present | Lob | Right | Upper | 6.0 | Alive | - | 1.6 | 1.6 | Solid | INV-1 | 1 | 1 |
| 549 | 66 | Male | Present | Lob | Right | Upper | 8.3 | Alive | - | 1.0 | 0.6 | Papillary | INV-1 | 0 | 0 |
| 550 | 66 | Male | Present | Seg | Left | Upper | 4.0 | Alive | - | 1.8 | 0.4 | Lepidic (MIA) | INV-1 | 0 | 0 |
| 551 | 66 | Male | Present | Wed | Left | Upper | 5.8 | Alive | - | 1.1 | 0.1 | Lepidic (MIA) | INV-2 | 0 | 0 |
| 552 | 66 | Male | Present | Wed | Right | Lower | 6.4 | Alive | - | 0.8 | 0.0 | AIS | AIS | 0 | 0 |
| 553 | 66 | Male | Present | Wed | Right | Upper | 7.3 | Alive | - | 1.3 | 0.8 | Lepidic | INV-1 | 0 | 0 |
| 554 | 67 | Female | Absent | Lob | Left | Upper | 4.4 | Alive | - | 1.3 | 0.5 | Lepidic (MIA) | INV-1 | 0 | 0 |
| 555 | 67 | Female | Absent | Lob | Left | Upper | 4.4 | Alive | - | 2.5 | 2.3 | Lepidic | INV-2 | 0 | 0 |
| 556 | 67 | Female | Absent | Lob | Right | Lower | 4.5 | Alive | - | 1.5 | 0.4 | Lepidic (MIA) | INV-2 | 0 | 0 |
| 557 | 67 | Female | Absent | Lob | Right | Lower | 5.5 | Alive | - | 2.3 | 1.9 | Lepidic | INV-1 | 0 | 0 |
| 558 | 67 | Female | Absent | Lob | Right | Middle | 6.9 | Alive | - | 1.2 | 0.0 | AIS | AIS | 0 | 0 |
| 559 | 67 | Female | Absent | Lob | Right | Upper | 8.5 | Alive | - | 2.2 | 2.2 | Lepidic | INV-1 | 0 | 0 |
| 560 | 67 | Female | Absent | Lob | Right | Upper | 10.3 | Alive | - | 1.5 | 0.6 | Papillary | INV-1 | 0 | 0 |
| 561 | 67 | Female | Absent | Lob | Right | Upper | 7.9 | Alive | - | 2.2 | 0.2 | Lepidic (MIA) | INV-1 | 0 | 0 |
| 562 | 67 | Female | Absent | Lob | Right | Upper | 5.5 | Alive | - | 2.8 | 1.6 | Lepidic | INV-2 | 0 | 0 |
| 563 | 67 | Female | Absent | Lob | Right | Upper | 5.4 | Alive | - | 1.6 | 0.5 | Lepidic (MIA) | INV-2 | 0 | 0 |
| 564 | 67 | Female | Absent | Lob | Right | Upper | 5.2 | Alive | - | 2.2 | 2.2 | Papillary | INV-1 | 0 | 0 |
| 565 | 67 | Female | Absent | Seg | Right | Lower | 6.0 | Alive | - | 1.6 | 0.3 | Lepidic (MIA) | INV-2 | 0 | 0 |
| 566 | 67 | Female | Absent | Wed | Left | Upper | 5.9 | Alive | - | 1.8 | 0.0 | AIS | AIS | 0 | 0 |
| 567 | 67 | Female | Absent | Wed | Left | Upper | 5.9 | Alive | - | 1.0 | 0.4 | Lepidic (MIA) | INV-1 | 0 | 0 |
| 568 | 67 | Female | Absent | Wed | Right | Lower | 4.5 | Alive | - | 2.0 | 0.1 | Lepidic (MIA) | INV-1 | 0 | 0 |
| 569 | 67 | Female | Absent | Wed | Right | Upper | 6.4 | Alive | - | 1.8 | 0.0 | AIS | AIS | 0 | 0 |
| 570 | 67 | Female | Absent | Wed | Right | Upper | 5.2 | Alive | - | 0.9 | 0.4 | Lepidic (MIA) | INV-1 | 0 | 0 |
| 571 | 67 | Female | Present | Seg | Left | Upper | 1.0 | Dead | + | 2.2 | 2.2 | Papillary | INV-1 | 1 | 1 |
| 572 | 67 | Male | Absent | Lob | Left | Upper | 5.6 | Alive | - | 2.3 | 2.3 | Lepidic | INV-1 | 1 | 0 |
| 573 | 67 | Male | Absent | Lob | Right | Lower | 4.3 | Alive | - | 2.5 | 2.5 | Papillary | INV-1 | 0 | 0 |
| 574 | 67 | Male | Absent | Lob | Right | Upper | 8.2 | Alive | - | 2.2 | 2.2 | Acinar | INV-1 | 0 | 1 |
| 575 | 67 | Male | Absent | Seg | Right | Lower | 4.1 | Alive | - | 0.9 | 0.9 | Acinar | INV-1 | 1 | 1 |
| 576 | 67 | Male | Present | Lob | Left | Upper | 8.3 | Alive | - | 1.8 | 1.8 | Solid | INV-1 | 1 | 1 |
| 577 | 67 | Male | Present | Lob | Left | Upper | 5.2 | Alive | - | 1.5 | 1.5 | Solid | INV-1 | 0 | 0 |
| 578 | 67 | Male | Present | Lob | Left | Upper | 4.6 | Alive | - | 2.8 | 2.8 | Solid | INV-1 | 0 | 0 |
| 579 | 67 | Male | Present | Lob | Left | Upper | 4.5 | Alive | - | 2.0 | 2.0 | Papillary | INV-1 | 1 | 1 |
| 580 | 67 | Male | Present | Lob | Right | Upper | 4.4 | Alive | - | 1.6 | 1.4 | Papillary | INV-1 | 1 | 1 |
| 581 | 67 | Male | Present | Lob | Right | Upper | 4.5 | Alive | - | 2.6 | 1.4 | Lepidic | INV-1 | 0 | 0 |
| 582 | 67 | Male | Present | Lob | Right | Upper | 3.6 | Alive | - | 1.5 | 1.5 | Papillary | INV-1 | 1 | 1 |
| 583 | 67 | Male | Present | Lob | Right | Upper | 5.0 | Alive | - | 2.1 | 2.1 | Acinar | INV-1 | 0 | 0 |
| 584 | 67 | Male | Present | Seg | Right | Lower | 3.5 | Alive | - | 2.0 | 2.0 | Papillary | INV-1 | 1 | 1 |
| 585 | 67 | Male | Present | Wed | Left | Upper | 6.5 | Alive | - | 1.5 | 0.6 | Lepidic | INV-2 | 0 | 0 |
| 586 | 67 | Male | Present | Wed | Left | Upper | 5.2 | Alive | - | 1.5 | 0.1 | Lepidic (MIA) | INV-1 | 0 | 0 |
| 587 | 67 | Male |  | Lob | Left | Lower | 1.2 | Alive | + | 2.6 | 2.6 | Acinar | INV-1 | 1 | 1 |
| 588 | 67 | Male |  | Seg | Right | Lower | 9.7 | Alive | - | 1.8 | 0.8 | Lepidic | INV-1 | 0 | 0 |
| 589 | 67 | Male |  | Wed | Left | Lower | 2.3 | Alive | - | 1.2 | 0.4 | Lepidic (MIA) | INV-1 | 0 | 0 |
| 590 | 68 | Female | Absent | Lob | Left | Upper | 4.1 | Alive | - | 2.5 | 2.5 | Acinar | INV-1 | 1 | 1 |
| 591 | 68 | Female | Absent | Lob | Right | Lower | 6.8 | Alive | - | 1.8 | 1.8 | Lepidic | INV-1 | 0 | 0 |
| 592 | 68 | Female | Absent | Lob | Right | Lower | 4.6 | Alive | - | 3.0 | 0.2 | Lepidic (MIA) | INV-1 | 0 | 0 |
| 593 | 68 | Female | Absent | Lob | Right | Middle | 4.5 | Alive | - | 1.8 | 1.8 | Papillary | INV-1 | 0 | 0 |
| 594 | 68 | Female | Absent | Lob | Right | Middle | 8.2 | Alive | - | 1.6 | 1.0 | Lepidic | INV-1 | 0 | 0 |
| 595 | 68 | Female | Absent | Lob | Right | Upper | 7.8 | Alive | - | 1.9 | 0.0 | AIS | AIS | 0 | 0 |
| 596 | 68 | Female | Absent | Lob | Right | Upper | 9.5 | Alive | - | 1.6 | 1.5 | Lepidic | INV-1 | 0 | 0 |
| 597 | 68 | Female | Absent | Lob | Right | Upper | 5.2 | Dead | - | 2.1 | 0.7 | Lepidic | INV-1 | 0 | 0 |
| 598 | 68 | Female | Absent | Lob | Right | Upper | 5.6 | Alive | - | 1.6 | 0.0 | AIS | AIS | 0 | 0 |
| 599 | 68 | Female | Absent | Lob | Right | Upper | 5.6 | Alive | - | 2.9 | 0.0 | AIS | AIS | 0 | 0 |
| 600 | 68 | Female | Absent | Lob | Right | Upper | 5.0 | Alive | - | 1.2 | 1.1 | Lepidic | INV-2 | 0 | 0 |
| 601 | 68 | Female | Absent | Lob | Right | Upper | 4.4 | Alive | - | 2.5 | 0.4 | Lepidic (MIA) | INV-1 | 0 | 0 |
| 602 | 68 | Female | Absent | Lob | Right | Upper | 4.1 | Alive | - | 2.4 | 2.4 | Lepidic | INV-1 | 0 | 0 |
| 603 | 68 | Female | Absent | Lob | Right | Upper | 5.3 | Alive | - | 1.8 | 1.8 | Papillary | INV-1 | 1 | 0 |
| 604 | 68 | Female | Absent | Seg | Right | Upper | 6.2 | Alive | - | 2.0 | 0.1 | Lepidic (MIA) | INV-1 | 0 | 0 |
| 605 | 68 | Female | Absent | Wed | Left | Upper | 4.0 | Alive | + | 1.5 | 0.9 | Papillary | INV-1 | 0 | 1 |
| 606 | 68 | Female | Absent | Wed | Left | Upper | 4.4 | Alive | - | 1.5 | 0.3 | Lepidic (MIA) | INV-2 | 0 | 0 |
| 607 | 68 | Female | Absent | Wed | Right | Upper | 5.0 | Alive | - | 1.3 | 0.0 | AIS | AIS | 0 | 0 |
| 608 | 68 | Female | Present | Lob | Left | Lower | 5.4 | Alive | - | 2.3 | 1.3 | Lepidic | INV-2 | 0 | 0 |
| 609 | 68 | Female | Present | Lob | Right | Upper | 6.0 | Alive | - | 1.5 | 1.0 | Papillary | INV-1 | 0 | 0 |
| 610 | 68 | Female | Present | Lob | Right | Upper | 3.6 | Alive | - | 2.1 | 2.1 | Micropapillary | INV-1 | 1 | 0 |
| 611 | 68 | Female | Present | Lob | Right | Upper | 4.0 | Alive | - | 2.8 | 2.8 | Variant | INV-1 | 0 | 1 |
| 612 | 68 | Female | Present | Lob | Right | Upper | 5.8 | Alive | - | 2.5 | 1.2 | Lepidic | INV-2 | 0 | 0 |
| 613 | 68 | Female | Present | Seg | Left | Lower | 4.6 | Alive | - | 1.5 | 0.7 | Lepidic | INV-2 | 0 | 0 |
| 614 | 68 | Female | Present | Wed | Left | Upper | 5.6 | Alive | - | 1.3 | 0.2 | Lepidic (MIA) | INV-1 | 0 | 0 |
| 615 | 68 | Female | Present | Wed | Right | Lower | 4.0 | Alive | - | 0.8 | 0.0 | AIS | AIS | 0 | 0 |
| 616 | 68 | Female | Present | Wed | Right | Upper | 6.4 | Alive | - | 1.9 | 0.6 | Lepidic | INV-2 | 0 | 0 |
| 617 | 68 | Male | Absent | Wed | Right | Lower | 1.9 | Alive | + | 0.8 | 0.8 | Papillary | INV-1 | 0 | 1 |
| 618 | 68 | Male | Present | Lob | Left | Lower | 6.2 | Alive | - | 2.9 | 2.5 | Papillary | INV-1 | 0 | 0 |
| 619 | 68 | Male | Present | Lob | Left | Upper | 8.8 | Alive | - | 1.9 | 1.0 | Papillary | INV-1 | 1 | 1 |
| 620 | 68 | Male | Present | Lob | Left | Upper | 4.9 | Alive | - | 2.0 | 0.0 | AIS | AIS | 0 | 0 |
| 621 | 68 | Male | Present | Lob | Right | Lower | 10.8 | Alive | - | 1.2 | 1.2 | Papillary | INV-1 | 1 | 0 |
| 622 | 68 | Male | Present | Lob | Right | Lower | 8.0 | Alive | - | 1.8 | 1.8 | Solid | INV-1 | 0 | 1 |
| 623 | 68 | Male | Present | Lob | Right | Lower | 5.5 | Alive | - | 2.5 | 1.0 | Lepidic | INV-1 | 0 | 0 |
| 624 | 68 | Male | Present | Wed | Left | Lower | 5.6 | Alive | - | 1.4 | 0.6 | Papillary | INV-1 | 0 | 0 |
| 625 | 68 | Male | Present | Wed | Right | Lower | 5.0 | Alive | - | 1.1 | 0.0 | AIS | AIS | 0 | 0 |
| 626 | 69 | Female | Absent | Lob | Left | Lower | 6.2 | Alive | - | 2.1 | 0.7 | Lepidic | INV-1 | 0 | 0 |
| 627 | 69 | Female | Absent | Lob | Left | Lower | 6.3 | Alive | - | 1.4 | 1.2 | Papillary | INV-1 | 0 | 0 |
| 628 | 69 | Female | Absent | Lob | Left | Lower | 3.8 | Alive | - | 1.7 | 1.7 | Papillary | INV-1 | 0 | 0 |
| 629 | 69 | Female | Absent | Lob | Left | Upper | 5.8 | Alive | - | 1.5 | 1.5 | Lepidic | INV-1 | 0 | 0 |
| 630 | 69 | Female | Absent | Lob | Left | Upper | 8.2 | Alive | - | 1.6 | 0.6 | Lepidic | INV-2 | 0 | 0 |
| 631 | 69 | Female | Absent | Lob | Right | Lower | 6.0 | Alive | - | 2.8 | 2.8 | Papillary | INV-1 | 0 | 0 |
| 632 | 69 | Female | Absent | Lob | Right | Lower | 9.3 | Alive | - | 2.7 | 2.7 | Lepidic | INV-1 | 0 | 0 |
| 633 | 69 | Female | Absent | Lob | Right | Middle | 5.2 | Alive | - | 1.2 | 0.6 | Lepidic | INV-1 | 0 | 0 |
| 634 | 69 | Female | Absent | Lob | Right | Upper | 6.4 | Alive | - | 1.6 | 0.4 | Lepidic (MIA) | INV-1 | 0 | 0 |
| 635 | 69 | Female | Absent | Lob | Right | Upper | 8.1 | Alive | - | 2.2 | 0.2 | Lepidic (MIA) | INV-2 | 0 | 0 |
| 636 | 69 | Female | Absent | Lob | Right | Upper | 5.9 | Alive | - | 1.9 | 1.0 | Lepidic | INV-1 | 0 | 0 |
| 637 | 69 | Female | Absent | Lob | Right | Upper | 5.0 | Alive | - | 2.6 | 0.0 | AIS | AIS | 0 | 0 |
| 638 | 69 | Female | Absent | Lob | Right | Upper | 6.1 | Alive | - | 2.5 | 0.1 | Lepidic (MIA) | INV-1 | 0 | 0 |
| 639 | 69 | Female | Absent | Wed | Right | Upper | 6.0 | Alive | - | 1.0 | 0.6 | Lepidic | INV-2 | 0 | 0 |
| 640 | 69 | Female | Present | Lob | Left | Lower | 7.8 | Alive | - | 1.7 | 1.7 | Lepidic | INV-1 | 0 | 0 |
| 641 | 69 | Female | Present | Lob | Left | Lower | 6.0 | Alive | - | 1.1 | 1.1 | Papillary | INV-1 | 0 | 0 |
| 642 | 69 | Female | Present | Lob | Right | Lower | 6.7 | Alive | - | 2.7 | 2.7 | Lepidic | INV-1 | 0 | 0 |
| 643 | 69 | Female | Present | Lob | Right | Lower | 5.8 | Alive | - | 2.6 | 0.9 | Lepidic | INV-2 | 0 | 0 |
| 644 | 69 | Female |  | Lob | Right | Upper | 8.1 | Alive | - | 1.5 | 0.8 | Lepidic | INV-1 | 0 | 0 |
| 645 | 69 | Male | Absent | Lob | Right | Upper | 6.7 | Alive | - | 0.9 | 0.7 | Lepidic | INV-1 | 0 | 0 |
| 646 | 69 | Male | Absent | Lob | Right | Upper | 11.3 | Alive | - | 2.1 | 2.1 | Lepidic | INV-1 | 0 | 0 |
| 647 | 69 | Male | Absent | Lob | Right | Upper | 4.6 | Alive | - | 1.9 | 1.2 | Acinar | INV-1 | 0 | 0 |
| 648 | 69 | Male | Absent | Seg | Left | Upper | 5.5 | Alive | - | 1.1 | 0.0 | AIS | AIS | 0 | 0 |
| 649 | 69 | Male | Absent | Wed | Left | Lower | 5.0 | Alive | - | 0.9 | 0.0 | AIS | AIS | 0 | 0 |
| 650 | 69 | Male | Absent | Wed | Left | Upper | 6.4 | Alive | - | 1.6 | 0.0 | AIS | AIS | 0 | 0 |
| 651 | 69 | Male | Present | Lob | Left | Lower | 2.3 | Alive | - | 1.6 | 1.6 | Solid | INV-1 | 0 | 1 |
| 652 | 69 | Male | Present | Lob | Left | Lower | 9.0 | Alive | - | 2.8 | 1.5 | Papillary | INV-1 | 1 | 1 |
| 653 | 69 | Male | Present | Lob | Left | Lower | 5.3 | Alive | - | 2.2 | 2.2 | Papillary | INV-1 | 0 | 0 |
| 654 | 69 | Male | Present | Lob | Left | Upper | 6.2 | Alive | - | 1.5 | 0.9 | Lepidic | INV-1 | 0 | 0 |
| 655 | 69 | Male | Present | Lob | Left | Upper | 4.3 | Alive | - | 2.0 | 2.0 | Lepidic | INV-1 | 0 | 0 |
| 656 | 69 | Male | Present | Lob | Right | Upper | 5.0 | Alive | - | 1.3 | 0.6 | Lepidic | INV-1 | 0 | 0 |
| 657 | 69 | Male | Present | Lob | Right | Upper | 0.0 | Alive | + | 2.2 | 2.2 | Papillary | INV-1 | 0 | 0 |
| 658 | 69 | Male | Present | Lob | Right | Upper | 4.6 | Alive | - | 1.7 | 0.6 | Lepidic | INV-1 | 0 | 0 |
| 659 | 69 | Male | Present | Lob | Right | Upper | 5.6 | Alive | - | 2.5 | 1.3 | Lepidic | INV-2 | 0 | 0 |
| 660 | 69 | Male | Present | Pneu | Left | Upper | 6.6 | Alive | - | 2.0 | 2.0 | Lepidic | INV-1 | 0 | 0 |
| 661 | 69 | Male | Present | Seg | Left | Upper | 3.4 | Alive | + | 2.8 | 2.8 | Papillary | INV-1 | 1 | 0 |
| 662 | 69 | Male | Present | Wed | Left | Upper | 4.0 | Alive | - | 0.7 | 0.7 | Solid | INV-1 | 0 | 0 |
| 663 | 69 | Male | Present | Wed | Right | Middle | 2.1 | Dead | - | 1.5 | 0.0 | AIS | AIS | 0 | 0 |
| 664 | 69 | Male | Present | Wed | Right | Middle | 1.4 | Dead | + | 1.2 | 1.2 | Solid | INV-1 | 1 | 1 |
| 665 | 69 | Male | Present | Wed | Right | Upper | 5.0 | Alive | - | 1.0 | 0.7 | Lepidic | INV-2 | 0 | 0 |
| 666 | 69 | Male | Present | Wed | Right | Upper | 5.5 | Alive | - | 2.3 | 1.0 | Lepidic | INV-2 | 0 | 0 |
| 667 | 69 | Male |  | Seg | Left | Upper | 5.0 | Alive | - | 1.0 | 1.0 | Acinar | INV-1 | 0 | 0 |
| 668 | 70 | Female | Absent | Lob | Left | Lower | 6.0 | Alive | - | 1.7 | 1.7 | Lepidic | INV-1 | 0 | 0 |
| 669 | 70 | Female | Absent | Lob | Left | Lower | 5.1 | Alive | - | 2.4 | 2.4 | Papillary | INV-1 | 0 | 0 |
| 670 | 70 | Female | Absent | Lob | Left | Upper | 6.9 | Alive | - | 2.1 | 2.1 | Lepidic | INV-1 | 0 | 0 |
| 671 | 70 | Female | Absent | Lob | Left | Upper | 5.9 | Alive | - | 2.9 | 2.6 | Lepidic | INV-1 | 0 | 0 |
| 672 | 70 | Female | Absent | Lob | Left | Upper | 8.1 | Alive | - | 2.2 | 0.2 | Lepidic (MIA) | INV-2 | 0 | 0 |
| 673 | 70 | Female | Absent | Lob | Left | Upper | 5.4 | Alive | - | 2.8 | 1.6 | Lepidic | INV-1 | 0 | 0 |
| 674 | 70 | Female | Absent | Lob | Right | Lower | 7.0 | Alive | - | 1.8 | 0.1 | Lepidic (MIA) | INV-2 | 0 | 0 |
| 675 | 70 | Female | Absent | Lob | Right | Lower | 8.6 | Alive | - | 2.8 | 1.2 | Lepidic | INV-1 | 0 | 0 |
| 676 | 70 | Female | Absent | Lob | Right | Lower | 5.3 | Alive | - | 2.3 | 2.3 | Papillary | INV-1 | 0 | 0 |
| 677 | 70 | Female | Absent | Lob | Right | Middle | 5.8 | Alive | - | 2.5 | 1.4 | Lepidic | INV-1 | 0 | 0 |
| 678 | 70 | Female | Absent | Lob | Right | Upper | 8.4 | Alive | - | 2.3 | 0.8 | Lepidic | INV-2 | 0 | 0 |
| 679 | 70 | Female | Absent | Lob | Right | Upper | 5.8 | Alive | - | 2.4 | 0.2 | Lepidic (MIA) | INV-1 | 0 | 0 |
| 680 | 70 | Female | Absent | Lob | Right | Upper | 4.5 | Alive | - | 2.5 | 0.4 | Lepidic (MIA) | INV-2 | 0 | 0 |
| 681 | 70 | Female | Absent | Lob | Right | Upper | 5.7 | Alive | - | 2.5 | 1.7 | Lepidic | INV-1 | 0 | 0 |
| 682 | 70 | Female | Absent | Lob | Right | Upper | 3.5 | Alive | - | 1.0 | 0.0 | AIS | AIS | 0 | 0 |
| 683 | 70 | Female | Absent | Lob | Right | Upper | 5.6 | Alive | - | 1.3 | 1.1 | Lepidic | INV-2 | 0 | 0 |
| 684 | 70 | Female | Absent | Seg | Left | Upper | 4.3 | Alive | - | 2.2 | 1.2 | Acinar | INV-1 | 0 | 0 |
| 685 | 70 | Female | Absent | Seg | Left | Upper | 5.5 | Alive | - | 1.5 | 0.7 | Acinar | INV-1 | 0 | 0 |
| 686 | 70 | Female | Absent | Seg | Right | Lower | 3.9 | Alive | - | 1.4 | 0.0 | AIS | AIS | 0 | 0 |
| 687 | 70 | Female | Absent | Wed | Left | Lower | 5.5 | Alive | - | 1.8 | 0.1 | Lepidic (MIA) | INV-1 | 0 | 0 |
| 688 | 70 | Female | Absent | Wed | Right | Upper | 4.8 | Alive | - | 1.3 | 0.2 | Lepidic (MIA) | INV-1 | 0 | 0 |
| 689 | 70 | Female | Present | Lob | Right | Lower | 10.2 | Alive | - | 1.5 | 0.6 | Acinar | INV-1 | 1 | 1 |
| 690 | 70 | Female | Present | Lob | Right | Lower | 5.2 | Alive | - | 2.1 | 0.7 | Lepidic | INV-1 | 0 | 0 |
| 691 | 70 | Female | Present | Seg | Right | Middle | 11.2 | Alive | - | 1.5 | 0.0 | AIS | AIS | 0 | 0 |
| 692 | 70 | Female | Present | Wed | Right | Upper | 5.0 | Alive | - | 1.9 | 0.0 | AIS | AIS | 0 | 0 |
| 693 | 70 | Male | Absent | Lob | Left | Upper | 1.3 | Alive | - | 2.0 | 2.0 | Papillary | INV-1 | 1 | 1 |
| 694 | 70 | Male | Present | Lob | Left | Lower | 8.3 | Alive | - | 1.8 | 1.8 | Papillary | INV-1 | 1 | 1 |
| 695 | 70 | Male | Present | Lob | Left | Lower | 5.7 | Alive | - | 1.7 | 1.7 | Acinar | INV-1 | 0 | 0 |
| 696 | 70 | Male | Present | Lob | Left | Upper | 5.7 | Alive | - | 1.5 | 0.0 | AIS | AIS | 0 | 0 |
| 697 | 70 | Male | Present | Lob | Right | Lower | 1.8 | Dead | - | 3.0 | 0.6 | Lepidic | INV-1 | 1 | 1 |
| 698 | 70 | Male | Present | Lob | Right | Upper | 6.6 | Alive | - | 1.5 | 0.4 | Lepidic (MIA) | INV-1 | 0 | 0 |
| 699 | 70 | Male | Present | Lob | Right | Upper | 6.5 | Alive | - | 2.0 | 1.0 | Lepidic | INV-2 | 0 | 0 |
| 700 | 70 | Male | Present | Lob | Right | Upper | 5.5 | Alive | - | 1.5 | 0.8 | Lepidic | INV-1 | 0 | 0 |
| 701 | 70 | Male | Present | Lob | Right | Upper | 9.0 | Alive | - | 1.7 | 0.3 | Lepidic (MIA) | INV-1 | 0 | 0 |
| 702 | 70 | Male | Present | Lob | Right | Upper | 4.6 | Alive | - | 1.4 | 1.4 | Lepidic | INV-1 | 0 | 1 |
| 703 | 70 | Male | Present | Lob | Right | Upper | 4.5 | Alive | - | 1.0 | 1.0 | Papillary | INV-1 | 0 | 0 |
| 704 | 70 | Male | Present | Lob | Right | Upper | 3.6 | Alive | - | 2.5 | 1.4 | Lepidic | INV-1 | 0 | 0 |
| 705 | 70 | Male | Present | Seg | Right | Lower | 4.9 | Alive | - | 1.2 | 1.2 | Acinar | INV-1 | 1 | 1 |
| 706 | 70 | Male | Present | Wed | Right | Upper | 6.4 | Alive | - | 1.2 | 1.2 | Acinar | INV-1 | 0 | 0 |
| 707 | 70 | Male | Present | Wed | Right | Upper | 5.1 | Alive | - | 1.8 | 1.8 | Lepidic | INV-1 | 0 | 0 |
| 708 | 71 | Female | Absent | Lob | Left | Lower | 11.1 | Alive | - | 1.5 | 1.5 | Lepidic | INV-1 | 0 | 0 |
| 709 | 71 | Female | Absent | Lob | Left | Lower | 6.0 | Alive | - | 1.3 | 1.3 | Lepidic | INV-1 | 0 | 0 |
| 710 | 71 | Female | Absent | Lob | Left | Upper | 5.7 | Alive | - | 2.7 | 2.7 | Lepidic | INV-1 | 0 | 0 |
| 711 | 71 | Female | Absent | Lob | Left | Upper | 5.6 | Alive | - | 2.8 | 2.8 | Lepidic | INV-1 | 0 | 0 |
| 712 | 71 | Female | Absent | Lob | Right | Lower | 11.1 | Alive | - | 1.7 | 1.7 | Solid | INV-1 | 1 | 1 |
| 713 | 71 | Female | Absent | Lob | Right | Lower | 5.6 | Alive | - | 2.0 | 0.9 | Lepidic | INV-1 | 0 | 0 |
| 714 | 71 | Female | Absent | Lob | Right | Lower | 8.2 | Alive | - | 2.6 | 1.0 | Lepidic | INV-1 | 0 | 0 |
| 715 | 71 | Female | Absent | Lob | Right | Lower | 6.1 | Alive | - | 2.8 | 0.6 | Lepidic | INV-1 | 0 | 0 |
| 716 | 71 | Female | Absent | Lob | Right | Lower | 1.4 | Alive | + | 2.5 | 0.4 | Lepidic (MIA) | INV-1 | 0 | 0 |
| 717 | 71 | Female | Absent | Lob | Right | Middle | 5.8 | Alive | - | 2.2 | 2.2 | Papillary | INV-1 | 0 | 0 |
| 718 | 71 | Female | Absent | Lob | Right | Upper | 6.6 | Alive | - | 1.9 | 0.0 | AIS | AIS | 0 | 0 |
| 719 | 71 | Female | Absent | Lob | Right | Upper | 7.7 | Alive | - | 1.5 | 0.3 | Lepidic (MIA) | INV-1 | 0 | 0 |
| 720 | 71 | Female | Absent | Lob | Right | Upper | 8.2 | Alive | - | 2.5 | 0.3 | Lepidic (MIA) | INV-1 | 0 | 0 |
| 721 | 71 | Female | Absent | Lob | Right | Upper | 4.0 | Alive | - | 3.0 | 3.0 | Lepidic | INV-1 | 0 | 0 |
| 722 | 71 | Female | Absent | Seg | Left | Lower | 5.3 | Alive | - | 1.8 | 0.1 | Lepidic (MIA) | INV-1 | 0 | 0 |
| 723 | 71 | Female | Absent | Seg | Left | Upper | 4.7 | Alive | - | 2.1 | 0.0 | AIS | AIS | 0 | 0 |
| 724 | 71 | Female | Absent | Seg | Right | Lower | 6.4 | Alive | - | 1.8 | 0.0 | AIS | AIS | 0 | 0 |
| 725 | 71 | Female | Absent | Seg | Right | Upper | 5.6 | Alive | - | 1.0 | 0.0 | AIS | AIS | 0 | 0 |
| 726 | 71 | Female | Absent | Wed | Right | Lower | 5.1 | Alive | - | 2.0 | 0.0 | AIS | AIS | 0 | 0 |
| 727 | 71 | Female | Present | Lob | Right | Upper | 6.3 | Alive | - | 1.2 | 0.0 | AIS | AIS | 0 | 0 |
| 728 | 71 | Female | Present | Lob | Right | Upper | 6.6 | Alive | - | 1.9 | 0.5 | Lepidic (MIA) | INV-2 | 0 | 0 |
| 729 | 71 | Male | Absent | Lob | Left | Upper | 5.8 | Alive | - | 2.3 | 0.4 | Lepidic (MIA) | INV-2 | 0 | 0 |
| 730 | 71 | Male | Absent | Seg | Left | Upper | 5.0 | Alive | - | 1.5 | 0.1 | Lepidic (MIA) | INV-1 | 0 | 0 |
| 731 | 71 | Male | Absent | Wed | Right | Upper | 4.9 | Alive | - | 1.5 | 0.3 | Lepidic (MIA) | INV-1 | 0 | 0 |
| 732 | 71 | Male | Absent | Wed | Right | Upper | 2.7 | Alive | - | 2.2 | 0.0 | AIS | AIS | 0 | 0 |
| 733 | 71 | Male | Present | Lob | Left | Lower | 9.5 | Alive | - | 2.6 | 0.5 | Lepidic (MIA) | INV-1 | 1 | 1 |
| 734 | 71 | Male | Present | Lob | Left | Lower | 4.1 | Alive | - | 2.9 | 2.9 | Papillary | INV-1 | 0 | 1 |
| 735 | 71 | Male | Present | Lob | Left | Upper | 4.7 | Alive | - | 2.7 | 2.7 | Papillary | INV-1 | 0 | 0 |
| 736 | 71 | Male | Present | Lob | Left | Upper | 6.0 | Alive | - | 2.6 | 2.6 | Micropapillary | INV-1 | 0 | 0 |
| 737 | 71 | Male | Present | Lob | Right | Lower | 4.0 | Alive | - | 1.8 | 1.8 | Micropapillary | INV-1 | 0 | 0 |
| 738 | 71 | Male | Present | Lob | Right | Upper | 1.6 | Alive | + | 2.5 | 2.5 | Solid | INV-1 | 0 | 1 |
| 739 | 71 | Male | Present | Lob | Right | Upper | 4.6 | Alive | - | 2.2 | 0.1 | Lepidic (MIA) | INV-1 | 0 | 0 |
| 740 | 71 | Male | Present | Lob | Right | Upper | 4.5 | Alive | - | 3.0 | 0.8 | Lepidic | INV-2 | 0 | 0 |
| 741 | 71 | Male | Present | Seg | Right | Upper | 7.1 | Alive | - | 1.2 | 0.0 | AIS | AIS | 0 | 0 |
| 742 | 71 | Male | Present | Wed | Right | Lower | 5.2 | Alive | - | 0.8 | 0.2 | Lepidic (MIA) | INV-1 | 0 | 0 |
| 743 | 71 | Male | Present | Wed | Right | Middle | 6.0 | Alive | - | 1.5 | 0.1 | Lepidic (MIA) | INV-1 | 0 | 0 |
| 744 | 72 | Female | Absent | Lob | Left | Lower | 6.4 | Alive | - | 2.4 | 2.4 | Lepidic | INV-1 | 0 | 0 |
| 745 | 72 | Female | Absent | Lob | Left | Lower | 4.1 | Alive | - | 2.4 | 1.5 | Lepidic | INV-1 | 0 | 1 |
| 746 | 72 | Female | Absent | Lob | Left | Lower | 4.6 | Alive | - | 1.6 | 1.6 | Variant | INV-1 | 0 | 0 |
| 747 | 72 | Female | Absent | Lob | Left | Upper | 2.6 | Alive | + | 2.3 | 2.3 | Acinar | INV-1 | 1 | 1 |
| 748 | 72 | Female | Absent | Lob | Left | Upper | 7.6 | Alive | - | 2.3 | 0.3 | Lepidic (MIA) | INV-2 | 0 | 0 |
| 749 | 72 | Female | Absent | Lob | Left | Upper | 6.9 | Alive | - | 2.9 | 0.9 | Lepidic | INV-1 | 0 | 0 |
| 750 | 72 | Female | Absent | Lob | Left | Upper | 5.5 | Alive | - | 2.5 | 2.5 | Lepidic | INV-1 | 0 | 0 |
| 751 | 72 | Female | Absent | Lob | Right | Lower | 5.9 | Alive | - | 2.6 | 2.6 | Lepidic | INV-1 | 1 | 0 |
| 752 | 72 | Female | Absent | Lob | Right | Lower | 5.0 | Alive | - | 1.0 | 1.0 | Papillary | INV-1 | 0 | 1 |
| 753 | 72 | Female | Absent | Lob | Right | Lower | 5.3 | Alive | - | 2.2 | 2.2 | Papillary | INV-1 | 0 | 1 |
| 754 | 72 | Female | Absent | Lob | Right | Upper | 1.2 | Alive | - | 1.6 | 1.6 | Papillary | INV-1 | 1 | 1 |
| 755 | 72 | Female | Absent | Lob | Right | Upper | 5.8 | Alive | - | 2.0 | 0.9 | Lepidic | INV-2 | 0 | 0 |
| 756 | 72 | Female | Absent | Lob | Right | Upper | 2.4 | Alive | - | 2.4 | 1.7 | Lepidic | INV-2 | 0 | 0 |
| 757 | 72 | Female | Absent | Lob | Right | Upper | 6.1 | Alive | - | 2.3 | 2.3 | Papillary | INV-1 | 0 | 0 |
| 758 | 72 | Female | Absent | Lob | Right | Upper | 5.6 | Alive | - | 1.7 | 1.2 | Lepidic | INV-2 | 0 | 0 |
| 759 | 72 | Female | Absent | Seg | Right | Lower | 4.5 | Alive | - | 1.5 | 0.7 | Lepidic | INV-2 | 0 | 0 |
| 760 | 72 | Female | Absent | Seg | Right | Upper | 5.2 | Alive | - | 1.2 | 0.2 | Lepidic (MIA) | INV-1 | 0 | 0 |
| 761 | 72 | Female | Absent | Wed | Left | Lower | 1.0 | Alive | + | 1.2 | 1.2 | Papillary | INV-1 | 0 | 0 |
| 762 | 72 | Female | Absent | Wed | Left | Upper | 4.8 | Alive | - | 0.9 | 0.4 | Lepidic (MIA) | INV-1 | 0 | 0 |
| 763 | 72 | Female | Absent | Wed | Right | Lower | 7.4 | Alive | - | 1.2 | 0.0 | AIS | AIS | 0 | 0 |
| 764 | 72 | Female | Absent | Wed | Right | Middle | 7.0 | Alive | - | 1.3 | 0.0 | AIS | AIS | 0 | 0 |
| 765 | 72 | Female | Present | Lob | Left | Lower | 5.9 | Alive | - | 1.8 | 1.0 | Lepidic | INV-1 | 0 | 0 |
| 766 | 72 | Female | Present | Lob | Left | Upper | 4.3 | Alive | - | 1.1 | 0.9 | Lepidic | INV-1 | 0 | 0 |
| 767 | 72 | Female | Present | Lob | Left | Upper | 6.2 | Alive | - | 3.0 | 1.8 | Papillary | INV-1 | 0 | 0 |
| 768 | 72 | Female | Present | Seg | Left | Upper | 6.2 | Alive | - | 1.4 | 1.4 | Papillary | INV-1 | 0 | 0 |
| 769 | 72 | Male | Present | Lob | Left | Lower | 9.4 | Alive | - | 2.5 | 0.4 | Lepidic (MIA) | INV-1 | 0 | 0 |
| 770 | 72 | Male | Present | Lob | Left | Upper | 7.4 | Alive | - | 2.1 | 0.8 | Acinar | INV-1 | 0 | 1 |
| 771 | 72 | Male | Present | Lob | Left | Upper | 6.0 | Alive | - | 1.9 | 0.9 | Lepidic | INV-2 | 0 | 0 |
| 772 | 72 | Male | Present | Lob | Left | Upper | 4.9 | Alive | - | 2.2 | 2.2 | Acinar | INV-1 | 1 | 1 |
| 773 | 72 | Male | Present | Lob | Right | Lower | 6.2 | Alive | - | 1.2 | 0.9 | Acinar | INV-1 | 0 | 0 |
| 774 | 72 | Male | Present | Lob | Right | Lower | 3.4 | Alive | - | 1.7 | 0.9 | Lepidic | INV-1 | 0 | 0 |
| 775 | 72 | Male | Present | Lob | Right | Middle | 5.8 | Alive | - | 1.2 | 1.2 | Papillary | INV-1 | 0 | 0 |
| 776 | 72 | Male | Present | Lob | Right | Upper | 7.0 | Alive | - | 1.2 | 0.6 | Lepidic | INV-2 | 0 | 0 |
| 777 | 72 | Male | Present | Lob | Right | Upper | 8.9 | Alive | - | 1.8 | 0.2 | Lepidic (MIA) | INV-1 | 0 | 0 |
| 778 | 72 | Male | Present | Lob | Right | Upper | 6.0 | Alive | - | 2.2 | 0.3 | Lepidic (MIA) | INV-1 | 0 | 0 |
| 779 | 72 | Male | Present | Wed | Right | Upper | 5.1 | Alive | - | 1.9 | 0.1 | Lepidic (MIA) | INV-2 | 0 | 0 |
| 780 | 73 | Female | Absent | Lob | Left | Lower | 4.1 | Alive | - | 2.3 | 2.3 | Acinar | INV-1 | 1 | 0 |
| 781 | 73 | Female | Absent | Lob | Left | Upper | 5.9 | Alive | - | 1.2 | 1.2 | Papillary | INV-1 | 0 | 0 |
| 782 | 73 | Female | Absent | Lob | Left | Upper | 7.6 | Alive | - | 2.4 | 1.2 | Lepidic | INV-1 | 1 | 0 |
| 783 | 73 | Female | Absent | Lob | Left | Upper | 4.7 | Alive | - | 1.4 | 1.4 | Papillary | INV-1 | 0 | 1 |
| 784 | 73 | Female | Absent | Lob | Right | Lower | 5.9 | Alive | - | 2.4 | 0.2 | Lepidic (MIA) | INV-1 | 0 | 0 |
| 785 | 73 | Female | Absent | Lob | Right | Lower | 5.0 | Alive | - | 1.9 | 1.4 | Papillary | INV-1 | 1 | 1 |
| 786 | 73 | Female | Absent | Lob | Right | Middle | 10.7 | Alive | - | 2.6 | 2.6 | Lepidic | INV-1 | 0 | 0 |
| 787 | 73 | Female | Absent | Lob | Right | Middle | 4.8 | Alive | + | 1.9 | 1.9 | Papillary | INV-1 | 1 | 0 |
| 788 | 73 | Female | Absent | Lob | Right | Middle | 2.4 | Alive | - | 2.2 | 0.4 | Lepidic (MIA) | INV-2 | 0 | 0 |
| 789 | 73 | Female | Absent | Lob | Right | Upper | 5.4 | Alive | - | 2.8 | 0.8 | Lepidic | INV-2 | 0 | 0 |
| 790 | 73 | Female | Absent | Lob | Right | Upper | 3.9 | Dead | - | 1.5 | 0.0 | AIS | AIS | 0 | 0 |
| 791 | 73 | Female | Absent | Lob | Right | Upper | 2.2 | Dead | + | 2.7 | 2.7 | Papillary | INV-1 | 0 | 0 |
| 792 | 73 | Female | Absent | Lob | Right | Upper | 5.1 | Alive | - | 2.0 | 0.7 | Lepidic | INV-2 | 0 | 0 |
| 793 | 73 | Female | Absent | Seg | Left | Lower | 5.3 | Alive | - | 1.9 | 0.0 | AIS | AIS | 0 | 0 |
| 794 | 73 | Female | Absent | Wed | Left | Lower | 9.4 | Alive | - | 1.9 | 1.0 | Lepidic | INV-1 | 0 | 0 |
| 795 | 73 | Female | Absent | Wed | Left | Lower | 4.5 | Alive | - | 0.6 | 0.0 | AIS | AIS | 0 | 0 |
| 796 | 73 | Female | Absent | Wed | Right | Lower | 2.8 | Alive | - | 1.9 | 1.9 | Papillary | INV-1 | 0 | 0 |
| 797 | 73 | Female | Present | Lob | Left | Upper | 7.7 | Alive | - | 1.8 | 0.2 | Lepidic (MIA) | INV-2 | 0 | 0 |
| 798 | 73 | Female | Present | Lob | Left | Upper | 3.7 | Alive | - | 2.6 | 0.1 | Lepidic (MIA) | INV-1 | 0 | 0 |
| 799 | 73 | Female | Present | Lob | Right | Upper | 4.9 | Alive | - | 2.1 | 2.1 | Lepidic | INV-1 | 0 | 0 |
| 800 | 73 | Female | Present | Lob | Right | Upper | 4.2 | Alive | - | 1.8 | 1.8 | Papillary | INV-1 | 0 | 0 |
| 801 | 73 | Female | Present | Wed | Left | Lower | 6.1 | Alive | - | 0.8 | 0.0 | AIS | AIS | 0 | 0 |
| 802 | 73 | Female | Present | Wed | Left | Lower | 5.5 | Alive | - | 1.2 | 0.2 | Lepidic (MIA) | INV-1 | 0 | 0 |
| 803 | 73 | Female |  | Lob | Left | Upper | 5.2 | Alive | - | 1.7 | 1.2 | Lepidic | INV-2 | 0 | 0 |
| 804 | 73 | Male | Absent | Lob | Left | Upper | 10.1 | Alive | - | 2.8 | 1.6 | Lepidic | INV-1 | 1 | 0 |
| 805 | 73 | Male | Absent | Lob | Right | Upper | 4.8 | Alive | + | 2.8 | 2.8 | Papillary | INV-1 | 0 | 0 |
| 806 | 73 | Male | Absent | Seg | Left | Lower | 5.7 | Alive | - | 2.0 | 1.8 | Acinar | INV-1 | 0 | 0 |
| 807 | 73 | Male | Present | Lob | Right | Lower | 5.3 | Alive | - | 1.8 | 1.8 | Solid | INV-1 | 0 | 1 |
| 808 | 73 | Male | Present | Seg | Left | Upper | 5.0 | Alive | - | 1.2 | 1.0 | Lepidic | INV-1 | 0 | 0 |
| 809 | 73 | Male | Present | Seg | Right | Lower | 6.2 | Alive | - | 1.5 | 1.5 | Lepidic | INV-1 | 0 | 0 |
| 810 | 73 | Male |  | Lob | Right | Lower | 4.9 | Alive | - | 1.7 | 1.7 | Variant | INV-1 | 0 | 0 |
| 811 | 74 | Female | Absent | Lob | Left | Lower | 6.1 | Alive | - | 1.5 | 0.0 | AIS | AIS | 0 | 0 |
| 812 | 74 | Female | Absent | Lob | Left | Upper | 1.1 | Alive | - | 2.4 | 1.5 | Lepidic | INV-2 | 0 | 0 |
| 813 | 74 | Female | Absent | Lob | Right | Middle | 1.4 | Alive | - | 2.5 | 2.5 | Lepidic | INV-1 | 0 | 0 |
| 814 | 74 | Female | Absent | Lob | Right | Middle | 4.4 | Alive | - | 2.1 | 0.6 | Lepidic | INV-1 | 0 | 0 |
| 815 | 74 | Female | Absent | Lob | Right | Upper | 6.2 | Alive | - | 2.0 | 2.0 | Papillary | INV-1 | 0 | 0 |
| 816 | 74 | Female | Absent | Lob | Right | Upper | 11.1 | Alive | - | 1.6 | 1.6 | Lepidic | INV-1 | 0 | 0 |
| 817 | 74 | Female | Absent | Lob | Right | Upper | 9.1 | Alive | - | 2.2 | 1.2 | Papillary | INV-1 | 1 | 1 |
| 818 | 74 | Female | Absent | Lob | Right | Upper | 8.3 | Alive | - | 1.7 | 1.0 | Papillary | INV-1 | 0 | 0 |
| 819 | 74 | Female | Absent | Lob | Right | Upper | 5.1 | Alive | - | 1.8 | 0.1 | Lepidic (MIA) | INV-1 | 0 | 0 |
| 820 | 74 | Female | Absent | Lob | Right | Upper | 4.9 | Alive | - | 1.5 | 0.0 | AIS | AIS | 0 | 0 |
| 821 | 74 | Female | Absent | Lob | Right | Upper | 5.2 | Alive | - | 2.8 | 0.0 | AIS | AIS | 0 | 0 |
| 822 | 74 | Female | Absent | Lob | Right | Upper | 5.6 | Alive | - | 1.9 | 0.5 | Lepidic (MIA) | INV-2 | 0 | 0 |
| 823 | 74 | Female | Absent | Lob | Right | Upper | 4.8 | Alive | - | 1.4 | 1.4 | Papillary | INV-1 | 0 | 0 |
| 824 | 74 | Female | Absent | Seg | Left | Lower | 4.1 | Alive | - | 1.8 | 1.8 | Variant | INV-1 | 0 | 0 |
| 825 | 74 | Female | Absent | Seg | Left | Upper | 5.2 | Alive | - | 1.2 | 0.0 | AIS | AIS | 0 | 0 |
| 826 | 74 | Female | Absent | Seg | Left | Upper | 2.8 | Alive | - | 2.4 | 1.5 | Papillary | INV-1 | 1 | 0 |
| 827 | 74 | Female | Absent | Wed | Right | Upper | 4.5 | Alive | - | 1.9 | 0.3 | Lepidic (MIA) | INV-1 | 0 | 0 |
| 828 | 74 | Female | Absent | Wed | Right | Upper | 5.1 | Alive | - | 0.9 | 0.4 | Lepidic (MIA) | INV-1 | 0 | 0 |
| 829 | 74 | Female | Present | Lob | Right | Lower | 8.1 | Alive | - | 1.7 | 0.4 | Lepidic (MIA) | INV-1 | 0 | 0 |
| 830 | 74 | Female | Present | Seg | Left | Lower | 5.7 | Alive | - | 1.8 | 0.0 | AIS | AIS | 0 | 0 |
| 831 | 74 | Female | Present | Seg | Left | Upper | 5.4 | Alive | - | 1.4 | 1.4 | Papillary | INV-1 | 0 | 0 |
| 832 | 74 | Female | Present | Wed | Right | Upper | 2.3 | Alive | - | 2.4 | 2.4 | Acinar | INV-1 | 1 | 0 |
| 833 | 74 | Female | Present | Wed | Right | Upper | 2.0 | Alive | - | 2.0 | 0.7 | Lepidic | INV-2 | 0 | 0 |
| 834 | 74 | Male | Absent | Lob | Left | Lower | 3.8 | Alive | - | 1.8 | 1.8 | Acinar | INV-1 | 0 | 0 |
| 835 | 74 | Male | Absent | Lob | Left | Upper | 8.0 | Alive | - | 1.7 | 0.0 | AIS | AIS | 0 | 0 |
| 836 | 74 | Male | Absent | Lob | Right | Lower | 6.5 | Alive | - | 1.2 | 1.2 | Solid | INV-1 | 0 | 1 |
| 837 | 74 | Male | Absent | Lob | Right | Upper | 6.5 | Alive | - | 1.2 | 0.7 | Lepidic | INV-2 | 0 | 0 |
| 838 | 74 | Male | Absent | Seg | Left | Lower | 6.0 | Alive | - | 1.4 | 1.0 | Papillary | INV-1 | 0 | 0 |
| 839 | 74 | Male | Absent | Seg | Right | Lower | 6.1 | Alive | - | 1.1 | 0.7 | Lepidic | INV-1 | 0 | 0 |
| 840 | 74 | Male | Present | Lob | Left | Upper | 11.0 | Alive | - | 2.2 | 2.2 | Papillary | INV-1 | 1 | 1 |
| 841 | 74 | Male | Present | Lob | Left | Upper | 9.9 | Alive | - | 1.7 | 0.6 | Lepidic | INV-1 | 0 | 0 |
| 842 | 74 | Male | Present | Lob | Left | Upper | 8.3 | Alive | - | 1.5 | 1.5 | Papillary | INV-1 | 0 | 0 |
| 843 | 74 | Male | Present | Lob | Right | Upper | 5.6 | Alive | - | 2.2 | 1.4 | Lepidic | INV-1 | 0 | 0 |
| 844 | 74 | Male | Present | Lob | Right | Upper | 6.0 | Alive | - | 2.1 | 0.1 | Lepidic (MIA) | INV-2 | 0 | 0 |
| 845 | 74 | Male | Present | Lob | Right | Upper | 3.5 | Alive | - | 1.0 | 0.1 | Lepidic (MIA) | INV-1 | 0 | 0 |
| 846 | 74 | Male | Present | Lob | Right | Upper | 4.6 | Alive | - | 1.3 | 0.6 | Lepidic | INV-2 | 0 | 0 |
| 847 | 74 | Male | Present | Lob | Right | Upper | 0.2 | Dead | + | 1.8 | 1.8 | Solid | INV-1 | 0 | 0 |
| 848 | 74 | Male | Present | Seg | Left | Upper | 8.8 | Alive | - | 1.8 | 0.0 | AIS | AIS | 0 | 0 |
| 849 | 74 | Male | Present | Seg | Left | Upper | 5.6 | Alive | - | 2.0 | 0.3 | Lepidic (MIA) | INV-1 | 0 | 0 |
| 850 | 74 | Male | Present | Seg | Right | Upper | 5.6 | Alive | - | 1.1 | 0.4 | Lepidic (MIA) | INV-1 | 0 | 0 |
| 851 | 74 | Male | Present | Wed | Right | Upper | 3.1 | Alive | + | 2.6 | 2.6 | Variant | INV-1 | 0 | 0 |
| 852 | 74 | Male |  | Seg | Left | Upper | 10.7 | Alive | - | 1.6 | 0.5 | Lepidic (MIA) | INV-1 | 0 | 0 |
| 853 | 75 | Female | Absent | Lob | Left | Lower | 6.0 | Alive | - | 1.9 | 0.8 | Lepidic | INV-1 | 0 | 0 |
| 854 | 75 | Female | Absent | Lob | Left | Lower | 6.0 | Alive | - | 0.7 | 0.4 | Lepidic (MIA) | INV-1 | 0 | 0 |
| 855 | 75 | Female | Absent | Lob | Left | Lower | 5.0 | Alive | - | 1.2 | 0.6 | Papillary | INV-1 | 0 | 0 |
| 856 | 75 | Female | Absent | Lob | Left | Lower | 4.7 | Alive | - | 1.0 | 1.0 | Papillary | INV-1 | 0 | 0 |
| 857 | 75 | Female | Absent | Lob | Left | Upper | 5.2 | Alive | - | 1.6 | 0.4 | Lepidic (MIA) | INV-2 | 0 | 0 |
| 858 | 75 | Female | Absent | Lob | Right | Lower | 3.9 | Alive | - | 1.7 | 0.0 | AIS | AIS | 0 | 0 |
| 859 | 75 | Female | Absent | Lob | Right | Upper | 5.7 | Alive | - | 1.7 | 1.7 | Papillary | INV-1 | 0 | 0 |
| 860 | 75 | Female | Absent | Lob | Right | Upper | 5.8 | Alive | - | 2.0 | 1.3 | Lepidic | INV-2 | 0 | 0 |
| 861 | 75 | Female | Absent | Lob | Right | Upper | 5.1 | Alive | - | 2.8 | 0.1 | Lepidic (MIA) | INV-1 | 0 | 0 |
| 862 | 75 | Female | Absent | Seg | Right | Lower | 9.2 | Alive | - | 1.5 | 1.4 | Acinar | INV-1 | 0 | 0 |
| 863 | 75 | Female | Absent | Wed | Left | Lower | 4.2 | Alive | + | 1.5 | 1.5 | Variant | INV-1 | 0 | 0 |
| 864 | 75 | Female | Absent | Wed | Left | Upper | 6.4 | Alive | - | 1.7 | 0.0 | AIS | AIS | 0 | 0 |
| 865 | 75 | Female | Absent | Wed | Left | Upper | 6.4 | Alive | - | 1.8 | 0.0 | AIS | AIS | 0 | 0 |
| 866 | 75 | Female | Absent | Wed | Left | Upper | 3.5 | Alive | - | 1.5 | 0.0 | AIS | AIS | 0 | 0 |
| 867 | 75 | Female | Absent | Wed | Right | Middle | 5.5 | Alive | - | 0.7 | 0.0 | AIS | AIS | 0 | 0 |
| 868 | 75 | Female | Absent | Wed | Right | Middle | 5.6 | Alive | - | 1.5 | 0.0 | AIS | AIS | 0 | 0 |
| 869 | 75 | Female | Absent | Wed | Right | Upper | 5.8 | Alive | - | 1.4 | 1.4 | Lepidic | INV-1 | 0 | 0 |
| 870 | 75 | Female | Absent | Wed | Right | Upper | 5.6 | Alive | - | 1.3 | 0.4 | Lepidic (MIA) | INV-1 | 0 | 0 |
| 871 | 75 | Female | Present | Lob | Left | Lower | 5.1 | Alive | - | 1.4 | 0.3 | Lepidic (MIA) | INV-1 | 0 | 0 |
| 872 | 75 | Female | Present | Lob | Left | Lower | 6.0 | Alive | - | 2.2 | 2.2 | Papillary | INV-1 | 0 | 0 |
| 873 | 75 | Female | Present | Seg | Left | Upper | 3.8 | Alive | - | 1.4 | 1.4 | Solid | INV-1 | 1 | 1 |
| 874 | 75 | Female |  | Lob | Right | Lower | 5.0 | Alive | - | 1.1 | 1.1 | Papillary | INV-1 | 0 | 0 |
| 875 | 75 | Male | Absent | Lob | Left | Upper | 6.0 | Alive | + | 2.6 | 2.6 | Papillary | INV-1 | 0 | 0 |
| 876 | 75 | Male | Absent | Lob | Left | Upper | 5.6 | Alive | - | 1.7 | 1.7 | Acinar | INV-1 | 0 | 1 |
| 877 | 75 | Male | Absent | Lob | Right | Upper | 7.0 | Alive | - | 2.6 | 0.3 | Lepidic (MIA) | INV-2 | 0 | 0 |
| 878 | 75 | Male | Absent | Lob | Right | Upper | 5.8 | Alive | - | 2.6 | 0.0 | AIS | AIS | 0 | 0 |
| 879 | 75 | Male | Absent | Seg | Right | Upper | 6.2 | Alive | + | 2.4 | 2.4 | Papillary | INV-1 | 1 | 1 |
| 880 | 75 | Male | Present | Lob | Left | Lower | 4.4 | Alive | - | 1.5 | 1.5 | Solid | INV-1 | 0 | 1 |
| 881 | 75 | Male | Present | Lob | Left | Lower | 2.2 | Alive | - | 1.5 | 1.5 | Papillary | INV-1 | 0 | 0 |
| 882 | 75 | Male | Present | Lob | Left | Upper | 5.7 | Alive | - | 2.8 | 2.8 | Papillary | INV-1 | 0 | 0 |
| 883 | 75 | Male | Present | Lob | Right | Middle | 4.2 | Alive | - | 0.6 | 0.0 | AIS | AIS | 0 | 0 |
| 884 | 75 | Male | Present | Lob | Right | Upper | 1.6 | Dead | - | 2.1 | 2.1 | Lepidic | INV-1 | 0 | 0 |
| 885 | 75 | Male | Present | Lob | Right | Upper | 9.0 | Alive | - | 1.8 | 0.0 | AIS | AIS | 0 | 0 |
| 886 | 75 | Male | Present | Lob | Right | Upper | 6.0 | Alive | - | 1.4 | 0.4 | Lepidic (MIA) | INV-1 | 0 | 0 |
| 887 | 75 | Male | Present | Seg | Left | Lower | 8.4 | Alive | - | 2.3 | 0.3 | Lepidic (MIA) | INV-1 | 0 | 0 |
| 888 | 75 | Male | Present | Seg | Left | Lower | 4.2 | Alive | - | 1.5 | 1.5 | Lepidic | INV-1 | 0 | 0 |
| 889 | 75 | Male | Present | Seg | Right | Lower | 4.0 | Alive | - | 1.4 | 0.8 | Lepidic | INV-1 | 0 | 0 |
| 890 | 75 | Male | Present | Seg | Right | Upper | 7.0 | Alive | - | 2.0 | 0.5 | Lepidic (MIA) | INV-2 | 0 | 0 |
| 891 | 75 | Male | Present | Wed | Left | Upper | 4.0 | Alive | - | 1.5 | 0.4 | Lepidic (MIA) | INV-2 | 0 | 0 |
| 892 | 75 | Male | Present | Wed | Left | Upper | 3.8 | Alive | - | 2.4 | 0.4 | Lepidic (MIA) | INV-1 | 0 | 0 |
| 893 | 75 | Male | Present | Wed | Right | Lower | 4.7 | Alive | - | 3.0 | 3.0 | Variant | INV-1 | 0 | 0 |
| 894 | 76 | Female | Absent | Lob | Left | Lower | 1.3 | Alive | - | 2.2 | 0.5 | Lepidic (MIA) | INV-1 | 0 | 0 |
| 895 | 76 | Female | Absent | Lob | Left | Lower | 4.6 | Alive | - | 2.5 | 2.2 | Papillary | INV-1 | 0 | 0 |
| 896 | 76 | Female | Absent | Lob | Left | Lower | 5.0 | Alive | - | 1.4 | 1.4 | Papillary | INV-1 | 0 | 0 |
| 897 | 76 | Female | Absent | Lob | Left | Upper | 7.5 | Alive | - | 1.8 | 1.8 | Papillary | INV-1 | 0 | 0 |
| 898 | 76 | Female | Absent | Lob | Left | Upper | 4.0 | Alive | - | 1.8 | 0.0 | AIS | AIS | 0 | 0 |
| 899 | 76 | Female | Absent | Lob | Right | Middle | 6.5 | Alive | - | 0.8 | 0.0 | AIS | AIS | 0 | 0 |
| 900 | 76 | Female | Absent | Lob | Right | Middle | 5.3 | Alive | - | 1.8 | 1.8 | Papillary | INV-1 | 0 | 0 |
| 901 | 76 | Female | Absent | Lob | Right | Upper | 8.6 | Alive | - | 1.8 | 1.6 | Lepidic | INV-2 | 0 | 0 |
| 902 | 76 | Female | Absent | Lob | Right | Upper | 3.4 | Alive | - | 2.6 | 1.1 | Lepidic | INV-1 | 0 | 0 |
| 903 | 76 | Female | Absent | Lob | Right | Upper | 1.8 | Dead | - | 1.2 | 1.2 | Acinar | INV-1 | 0 | 0 |
| 904 | 76 | Female | Absent | Lob | Right | Upper | 6.0 | Alive | - | 1.5 | 0.0 | AIS | AIS | 0 | 0 |
| 905 | 76 | Female | Absent | Lob | Right | Upper | 3.1 | Alive | - | 2.0 | 0.5 | Lepidic (MIA) | INV-1 | 0 | 0 |
| 906 | 76 | Female | Absent | Seg | Left | Upper | 6.4 | Alive | - | 1.5 | 1.0 | Lepidic | INV-2 | 0 | 0 |
| 907 | 76 | Female | Absent | Wed | Left | Lower | 3.8 | Alive | - | 3.0 | 0.0 | AIS | AIS | 0 | 0 |
| 908 | 76 | Female | Absent | Wed | Left | Lower | 4.5 | Alive | - | 0.9 | 0.4 | Lepidic (MIA) | INV-1 | 0 | 0 |
| 909 | 76 | Female | Absent | Wed | Right | Upper | 4.0 | Alive | - | 1.0 | 0.0 | AIS | AIS | 0 | 0 |
| 910 | 76 | Female | Present | Lob | Left | Lower | 1.4 | Alive | - | 2.1 | 2.1 | Papillary | INV-1 | 1 | 1 |
| 911 | 76 | Female | Present | Lob | Left | Upper | 4.5 | Alive | - | 1.3 | 1.3 | Papillary | INV-1 | 0 | 0 |
| 912 | 76 | Female | Present | Lob | Right | Lower | 3.4 | Alive | - | 1.7 | 0.0 | AIS | AIS | 0 | 0 |
| 913 | 76 | Female |  | Lob | Right | Middle | 4.8 | Alive | - | 1.6 | 0.3 | Lepidic (MIA) | INV-1 | 0 | 0 |
| 914 | 76 | Male | Absent | Lob | Right | Lower | 5.1 | Alive | - | 2.8 | 2.8 | Papillary | INV-1 | 0 | 0 |
| 915 | 76 | Male | Absent | Seg | Left | Lower | 10.2 | Alive | - | 1.5 | 0.1 | Lepidic (MIA) | INV-2 | 0 | 0 |
| 916 | 76 | Male | Absent | Seg | Right | Upper | 4.4 | Alive | - | 1.8 | 0.8 | Lepidic | INV-2 | 0 | 0 |
| 917 | 76 | Male | Present | Lob | Left | Upper | 8.1 | Alive | - | 2.0 | 0.8 | Lepidic | INV-2 | 0 | 0 |
| 918 | 76 | Male | Present | Lob | Right | Middle | 2.7 | Alive | - | 1.8 | 0.3 | Lepidic (MIA) | INV-1 | 0 | 0 |
| 919 | 76 | Male | Present | Lob | Right | Upper | 6.1 | Alive | - | 1.3 | 0.0 | AIS | AIS | 0 | 0 |
| 920 | 76 | Male | Present | Lob | Right | Upper | 2.8 | Dead | + | 2.5 | 2.5 | Papillary | INV-1 | 1 | 0 |
| 921 | 76 | Male | Present | Wed | Right | Lower | 3.7 | Alive | - | 1.5 | 0.6 | Lepidic | INV-1 | 0 | 0 |
| 922 | 76 | Male | Present | Wed | Right | Upper | 5.7 | Alive | - | 2 | 2.0 | Acinar | INV-1 | 1 | 1 |
| 923 | 76 | Male |  | Lob | Left | Upper | 5.3 | Alive | - | 1.6 | 1.5 | Papillary | INV-1 | 0 | 0 |
| 924 | 77 | Female | Absent | Lob | Left | Lower | 6.5 | Alive | - | 1.7 | 1.7 | Lepidic | INV-1 | 0 | 0 |
| 925 | 77 | Female | Absent | Lob | Right | Lower | 5.5 | Alive | - | 1.4 | 1.4 | Lepidic | INV-1 | 0 | 0 |
| 926 | 77 | Female | Absent | Lob | Right | Upper | 3.2 | Alive | - | 2.8 | 2.6 | Lepidic | INV-1 | 0 | 0 |
| 927 | 77 | Female | Absent | Lob | Right | Upper | 5.0 | Alive | - | 2.5 | 0.8 | Lepidic | INV-1 | 0 | 0 |
| 928 | 77 | Female | Absent | Seg | Left | Upper | 5.4 | Alive | - | 2.3 | 2.0 | Lepidic | INV-1 | 0 | 0 |
| 929 | 77 | Female | Absent | Seg | Left | Upper | 5.1 | Alive | - | 1.8 | 0.0 | AIS | AIS | 0 | 0 |
| 930 | 77 | Female | Absent | Wed | Right | Lower | 8.5 | Alive | - | 1.5 | 1.5 | Lepidic | INV-1 | 0 | 0 |
| 931 | 77 | Female | Absent | Wed | Right | Lower | 10.0 | Alive | - | 2.5 | 0.8 | Lepidic | INV-1 | 0 | 1 |
| 932 | 77 | Female | Absent | Wed | Right | Lower | 4.4 | Alive | - | 1.2 | 0.8 | Lepidic | INV-1 | 0 | 0 |
| 933 | 77 | Female | Absent | Wed | Right | Upper | 3.7 | Alive | - | 1.6 | 1.6 | Papillary | INV-1 | 0 | 0 |
| 934 | 77 | Female | Present | Lob | Right | Middle | 3.5 | Alive | - | 1.6 | 0.0 | AIS | AIS | 0 | 0 |
| 935 | 77 | Male | Absent | Lob | Right | Upper | 6.3 | Alive | - | 1.9 | 1.9 | Papillary | INV-1 | 0 | 0 |
| 936 | 77 | Male | Present | Lob | Left | Upper | 6.0 | Alive | - | 2.8 | 2.8 | Papillary | INV-1 | 0 | 0 |
| 937 | 77 | Male | Present | Lob | Left | Upper | 5.6 | Alive | - | 2.5 | 2.5 | Papillary | INV-1 | 0 | 0 |
| 938 | 77 | Male | Present | Lob | Right | Lower | 8.6 | Alive | - | 1.2 | 1.2 | Papillary | INV-1 | 0 | 0 |
| 939 | 77 | Male | Present | Lob | Right | Lower | 2.6 | Alive | - | 2.8 | 2.8 | Papillary | INV-1 | 1 | 1 |
| 940 | 77 | Male | Present | Lob | Right | Upper | 1.6 | Alive | - | 2.5 | 2.5 | Papillary | INV-1 | 1 | 0 |
| 941 | 77 | Male | Present | Lob | Right | Upper | 6.0 | Alive | - | 1.7 | 0.1 | Lepidic (MIA) | INV-2 | 0 | 0 |
| 942 | 77 | Male | Present | Lob | Right | Upper | 7.2 | Alive | - | 2.8 | 2.8 | Solid | INV-1 | 0 | 0 |
| 943 | 77 | Male | Present | Lob | Right | Upper | 6.2 | Alive | - | 1.9 | 0.5 | Lepidic (MIA) | INV-1 | 0 | 0 |
| 944 | 77 | Male | Present | Wed | Left | Upper | 6.0 | Alive | - | 1.0 | 0.0 | AIS | AIS | 0 | 0 |
| 945 | 77 | Male | Present | Wed | Right | Lower | 1.8 | Dead | + | 1.9 | 1.9 | Solid | INV-1 | 0 | 0 |
| 946 | 77 | Male | Present | Wed | Right | Lower | 6.5 | Alive | - | 0.8 | 0.0 | AIS | AIS | 0 | 0 |
| 947 | 77 | Male | Present | Wed | Right | Upper | 9.4 | Alive | - | 2.0 | 0.7 | Lepidic | INV-1 | 0 | 0 |
| 948 | 77 | Male | Present | Wed | Right | Upper | 5.0 | Alive | - | 0.8 | 0.8 | Acinar | INV-1 | 0 | 0 |
| 949 | 77 | Male | Present | Wed | Right | Upper | 2.6 | Dead | + | 1.7 | 1.7 | Solid | INV-1 | 0 | 0 |
| 950 | 77 | Male | Present | Wed | Right | Upper | 4.1 | Alive | - | 1.4 | 0.0 | AIS | AIS | 0 | 0 |
| 951 | 78 | Female | Absent | Lob | Left | Lower | 4.7 | Alive | - | 2.3 | 2.3 | Solid | INV-1 | 1 | 1 |
| 952 | 78 | Female | Absent | Lob | Left | Upper | 5.7 | Alive | - | 2.3 | 2.3 | Lepidic | INV-1 | 0 | 0 |
| 953 | 78 | Female | Absent | Lob | Left | Upper | 5.3 | Alive | - | 1.8 | 0.6 | Lepidic | INV-2 | 0 | 0 |
| 954 | 78 | Female | Absent | Lob | Right | Lower | 5.1 | Alive | - | 2.6 | 2.6 | Lepidic | INV-1 | 0 | 0 |
| 955 | 78 | Female | Absent | Lob | Right | Lower | 4.9 | Alive | - | 1.8 | 1.8 | Acinar | INV-1 | 0 | 0 |
| 956 | 78 | Female | Absent | Lob | Right | Middle | 5.0 | Alive | - | 2.6 | 0.2 | Lepidic (MIA) | INV-1 | 0 | 0 |
| 957 | 78 | Female | Absent | Lob | Right | Upper | 9.7 | Alive | - | 2.5 | 0.0 | AIS | AIS | 0 | 0 |
| 958 | 78 | Female | Absent | Lob | Right | Upper | 4.3 | Alive | + | 1.5 | 0.9 | Papillary | INV-1 | 0 | 1 |
| 959 | 78 | Female | Absent | Lob | Right | Upper | 4.9 | Alive | - | 1.9 | 0.2 | Lepidic (MIA) | INV-2 | 0 | 0 |
| 960 | 78 | Female | Absent | Seg | Left | Upper | 8.7 | Alive | - | 1.4 | 0.0 | AIS | AIS | 0 | 0 |
| 961 | 78 | Female | Absent | Wed | Right | Lower | 5.3 | Alive | - | 0.8 | 0.0 | AIS | AIS | 0 | 0 |
| 962 | 78 | Male | Present | Lob | Left | Upper | 5.1 | Alive | - | 2.5 | 2.5 | Acinar | INV-1 | 0 | 0 |
| 963 | 78 | Male | Present | Lob | Left | Upper | 5.5 | Alive | - | 1.3 | 0.6 | Acinar | INV-1 | 0 | 0 |
| 964 | 78 | Male | Present | Lob | Right | Upper | 5.5 | Alive | - | 1.5 | 0.9 | Lepidic | INV-1 | 0 | 0 |
| 965 | 78 | Male | Present | Lob | Right | Upper | 4.3 | Alive | - | 2.3 | 2.3 | Lepidic | INV-1 | 0 | 0 |
| 966 | 78 | Male | Present | Lob | Right | Upper | 4.0 | Alive | - | 1.7 | 1.7 | Solid | INV-1 | 0 | 1 |
| 967 | 78 | Male | Present | Seg | Left | Upper | 6.5 | Alive | - | 2.5 | 0.8 | Lepidic | INV-2 | 0 | 0 |
| 968 | 78 | Male | Present | Seg | Left | Upper | 4.1 | Alive | - | 2.5 | 2.5 | Lepidic | INV-1 | 0 | 0 |
| 969 | 78 | Male | Present | Seg | Right | Lower | 4.7 | Alive | - | 1.6 | 1.6 | Papillary | INV-1 | 1 | 0 |
| 970 | 78 | Male | Present | Wed | Left | Lower | 4.7 | Alive | - | 1.5 | 1.5 | Papillary | INV-1 | 0 | 0 |
| 971 | 78 | Male | Present | Wed | Left | Upper | 9.4 | Alive | - | 2.9 | 2.9 | Papillary | INV-1 | 1 | 1 |
| 972 | 79 | Female | Absent | Lob | Left | Upper | 6.6 | Alive | - | 2.1 | 2.1 | Lepidic | INV-1 | 0 | 0 |
| 973 | 79 | Female | Absent | Lob | Left | Upper | 6.1 | Alive | - | 2.6 | 0.0 | AIS | AIS | 0 | 0 |
| 974 | 79 | Female | Absent | Lob | Left | Upper | 5.0 | Alive | - | 2.0 | 0.0 | AIS | AIS | 0 | 0 |
| 975 | 79 | Female | Absent | Lob | Right | Middle | 4.8 | Alive | - | 2.0 | 1.1 | Papillary | INV-1 | 0 | 0 |
| 976 | 79 | Female | Absent | Lob | Right | Upper | 8.9 | Alive | - | 2.3 | 2.0 | Papillary | INV-1 | 0 | 0 |
| 977 | 79 | Female | Absent | Lob | Right | Upper | 4.6 | Alive | - | 2.2 | 0.1 | Lepidic (MIA) | INV-1 | 0 | 0 |
| 978 | 79 | Female | Absent | Lob | Right | Upper | 3.9 | Alive | - | 2.0 | 1.0 | Lepidic | INV-1 | 0 | 0 |
| 979 | 79 | Female | Absent | Seg | Left | Upper | 6.6 | Alive | - | 2.5 | 0.6 | Lepidic | INV-1 | 0 | 0 |
| 980 | 79 | Female | Absent | Seg | Right | Lower | 5.1 | Alive | - | 0.9 | 0.0 | AIS | AIS | 0 | 0 |
| 981 | 79 | Female | Absent | Seg | Right | Lower | 3.4 | Alive | - | 1.2 | 0.0 | AIS | AIS | 0 | 0 |
| 982 | 79 | Female | Absent | Wed | Right | Lower | 10.0 | Alive | - | 0.9 | 0.5 | Papillary (MIA) | INV-1 | 0 | 0 |
| 983 | 79 | Female | Present | Lob | Right | Upper | 8.3 | Alive | - | 2.5 | 0.9 | Lepidic | INV-1 | 0 | 0 |
| 984 | 79 | Female | Present | Wed | Left | Upper | 6.5 | Alive | - | 1.2 | 0.4 | Lepidic (MIA) | INV-2 | 0 | 0 |
| 985 | 79 | Female | Present | Wed | Right | Lower | 6.1 | Alive | - | 1.2 | 1.2 | Variant | INV-1 | 0 | 0 |
| 986 | 79 | Female |  | Lob | Left | Upper | 6.6 | Alive | - | 2.4 | 2.4 | Papillary | INV-1 | 0 | 0 |
| 987 | 79 | Male | Absent | Wed | Right | Lower | 5.5 | Alive | - | 1.8 | 1.8 | Variant | INV-1 | 0 | 0 |
| 988 | 79 | Male | Present | Lob | Left | Upper | 2.2 | Alive | - | 2.5 | 1.1 | Lepidic | INV-1 | 0 | 0 |
| 989 | 79 | Male | Present | Lob | Right | Lower | 3.7 | Alive | - | 0.8 | 0.8 | Lepidic | INV-1 | 0 | 0 |
| 990 | 79 | Male | Present | Lob | Right | Upper | 5.5 | Alive | - | 2.2 | 2.2 | Papillary | INV-1 | 1 | 1 |
| 991 | 79 | Male | Present | Seg | Left | Upper | 6.2 | Alive | - | 1.5 | 0.1 | Lepidic (MIA) | INV-2 | 0 | 0 |
| 992 | 79 | Male | Present | Seg | Right | Lower | 8.1 | Alive | - | 2.1 | 1.5 | Lepidic | INV-1 | 0 | 0 |
| 993 | 79 | Male | Present | Wed | Left | Upper | 5.9 | Alive | - | 2.1 | 0.7 | Lepidic | INV-1 | 0 | 0 |
| 994 | 79 | Male | Present | Wed | Right | Upper | 5.7 | Alive | - | 1.8 | 0.5 | Lepidic (MIA) | INV-1 | 0 | 0 |
| 995 | 80 | Female | Absent | Lob | Left | Lower | 3.1 | Alive | - | 1.9 | 1.9 | Acinar | INV-1 | 1 | 1 |
| 996 | 80 | Female | Absent | Lob | Right | Lower | 5.1 | Alive | - | 2.4 | 2.4 | Lepidic | INV-1 | 0 | 0 |
| 997 | 80 | Female | Absent | Lob | Right | Middle | 4.5 | Alive | - | 1.7 | 1.7 | Papillary | INV-1 | 0 | 1 |
| 998 | 80 | Female | Absent | Lob | Right | Upper | 4.2 | Alive | - | 2.8 | 1.3 | Lepidic | INV-1 | 0 | 0 |
| 999 | 80 | Female | Absent | Wed | Right | Lower | 5.6 | Alive | - | 2.1 | 1.0 | Lepidic | INV-2 | 0 | 0 |
| 1000 | 80 | Female | Absent | Wed | Right | Lower | 3.2 | Alive | - | 1.8 | 0.0 | AIS | AIS | 0 | 0 |
| 1001 | 80 | Female | Absent | Wed | Right | Upper | 4.5 | Alive | - | 1.8 | 1.8 | Lepidic | INV-1 | 0 | 0 |
| 1002 | 80 | Male | Absent | Lob | Right | Upper | 5.1 | Alive | - | 2.7 | 2.7 | Lepidic | INV-1 | 1 | 0 |
| 1003 | 80 | Male | Present | Lob | Left | Lower | 5.1 | Alive | - | 2.3 | 1.3 | Lepidic | INV-1 | 0 | 0 |
| 1004 | 80 | Male | Present | Lob | Left | Upper | 2.4 | Alive | - | 2.5 | 2.5 | Papillary | INV-1 | 0 | 1 |
| 1005 | 80 | Male | Present | Lob | Left | Upper | 4.1 | Alive | - | 2.7 | 0.8 | Lepidic | INV-1 | 0 | 0 |
| 1006 | 80 | Male |  | Wed | Left | Upper | 3.9 | Alive | - | 1.5 | 1.5 | Acinar | INV-1 | 0 | 0 |
| 1007 | 81 | Female | Absent | Lob | Left | Upper | 3.7 | Alive | - | 2.3 | 2.3 | Papillary | INV-1 | 0 | 0 |
| 1008 | 81 | Female | Absent | Wed | Left | Upper | 5.8 | Alive | - | 2.2 | 0.1 | Lepidic (MIA) | INV-1 | 0 | 0 |
| 1009 | 81 | Female | Absent | Wed | Right | Upper | 5.5 | Alive | - | 1.2 | 0.9 | Lepidic | INV-2 | 0 | 0 |
| 1010 | 81 | Male | Present | Lob | Left | Lower | 5.0 | Alive | - | 0.8 | 0.8 | Acinar | INV-1 | 1 | 1 |
| 1011 | 81 | Male | Present | Lob | Left | Upper | 5.5 | Alive | - | 1.4 | 0.4 | Lepidic (MIA) | INV-1 | 0 | 0 |
| 1012 | 81 | Male | Present | Lob | Left | Upper | 4.2 | Alive | - | 2.0 | 2.0 | Papillary | INV-1 | 1 | 1 |
| 1013 | 81 | Male | Present | Lob | Right | Upper | 5.0 | Alive | - | 1.9 | 1.9 | Lepidic | INV-1 | 0 | 0 |
| 1014 | 81 | Male | Present | Wed | Left | Upper | 5.4 | Alive | - | 2.5 | 2.5 | Papillary | INV-1 | 0 | 0 |
| 1015 | 81 | Male | Present | Wed | Right | Lower | 3.8 | Alive | - | 1.3 | 1.3 | Variant | INV-1 | 0 | 0 |
| 1016 | 82 | Female | Absent | Lob | Right | Upper | 7.2 | Alive | - | 2.4 | 2.0 | Acinar | INV-1 | 0 | 0 |
| 1017 | 82 | Female | Absent | Wed | Left | Lower | 4.3 | Alive | - | 1.2 | 0.8 | Lepidic | INV-1 | 0 | 0 |
| 1018 | 82 | Male | Present | Wed | Right | Upper | 5.0 | Alive | - | 1.0 | 0.5 | Lepidic (MIA) | INV-1 | 0 | 0 |
| 1019 | 83 | Female | Absent | Lob | Right | Lower | 5.8 | Alive | - | 2.9 | 2.9 | Papillary | INV-1 | 0 | 0 |
| 1020 | 83 | Female | Absent | Seg | Left | Upper | 5.5 | Alive | - | 1.5 | 0.8 | Lepidic | INV-1 | 0 | 0 |
| 1021 | 83 | Male | Present | Lob | Right | Upper | 4.9 | Alive | - | 1.5 | 1.5 | Papillary | INV-1 | 1 | 0 |
| 1022 | 83 | Male | Present | Seg | Left | Upper | 5.0 | Alive | - | 1.8 | 1.8 | Papillary | INV-1 | 0 | 0 |
| 1023 | 83 | Male | Present | Seg | Left | Upper | 4.8 | Alive | - | 2.5 | 0.1 | Lepidic (MIA) | INV-2 | 0 | 0 |
| 1024 | 83 | Male | Present | Wed | Right | Lower | 4.3 | Alive | - | 2.8 | 2.8 | Variant | INV-1 | 0 | 0 |
| 1025 | 84 | Male | Present | Lob | Right | Middle | 2.1 | Dead | - | 1.1 | 1.1 | Acinar | INV-1 | 1 | 0 |
| 1026 | 85 | Female | Absent | Lob | Right | Upper | 5.3 | Alive | - | 1.9 | 0.5 | Lepidic (MIA) | INV-1 | 0 | 0 |
| 1027 | 85 | Female | Present | Lob | Left | Upper | 3.8 | Alive | + | 1.6 | 1.6 | Acinar | INV-1 | 1 | 1 |
| 1028 | 85 | Male | Present | Wed | Left | Upper | 2.6 | Alive | - | 1.4 | 1.4 | Solid | INV-1 | 0 | 1 |
| 1029 | 85 | Male | Present | Wed | Right | Upper | 1.4 | Alive | - | 1.8 | 0.6 | Lepidic | INV-1 | 0 | 0 |
| 1030 | 86 | Female | Absent | Lob | Right | Upper | 5.0 | Alive | - | 2.4 | 1.5 | Lepidic | INV-1 | 0 | 0 |
| 1031 | 86 | Female | Present | Seg | Right | Lower | 4.7 | Alive | - | 1.7 | 0.3 | Lepidic (MIA) | INV-1 | 0 | 0 |
| 1032 | 86 | Female | Present | Wed | Left | Lower | 3.9 | Alive | - | 2.4 | 0.9 | Lepidic | INV-1 | 0 | 0 |

Lob, lobectomy; Seg, segmentectomy; Wed, wedge resection
